# Loss of Caveolin-1 and caveolae leads to increased cardiac cell stiffness and functional decline of the adult zebrafish heart

**DOI:** 10.1038/s41598-020-68802-9

**Published:** 2020-07-30

**Authors:** Dimitrios Grivas, Álvaro González-Rajal, Carlos Guerrero Rodríguez, Ricardo Garcia, José Luis de la Pompa

**Affiliations:** 1 Intercellular Signalling in Cardiovascular Development and Disease Laboratory, Centro Nacional de Investigaciones Cardiovasculares Carlos III (CNIC), Melchor Fernández Almagro 3, 28029 Madrid, Spain; 2 Ciber de Enfermedades Cardiovasculares, 28029 Madrid, Spain; 3 Cell Division Lab, ANZAC Research Institute, Gate 3, Hospital Road, Concord, NSW 2139 Australia; 4 Materials Science Factory, Instituto de Ciencia de Materiales de Madrid (ICMM), CSIC, 28049 Madrid, Spain

**Keywords:** Cell biology, Developmental biology, Cell proliferation, Differentiation, Experimental organisms, Organogenesis, Stem cells

## Abstract

Caveolin-1 is the main structural protein of caveolae, small membrane invaginations involved in signal transduction and mechanoprotection. Here, we generated *cav1-KO* zebrafish lacking Cav1 and caveolae, and investigated the impact of this loss on adult heart function and response to cryoinjury. We found that cardiac function was impaired in adult *cav1-KO* fish, which showed a significantly decreased ejection fraction and heart rate. Using atomic force microscopy, we detected an increase in the stiffness of epicardial cells and cells of the cortical zone lacking Cav1/caveolae. This loss of cardiac elasticity might explain the decreased cardiac contraction and function. Surprisingly, *cav1-KO* mutants were able to regenerate their heart after a cryoinjury but showed a transient decrease in cardiomyocyte proliferation.

## Introduction

Caveolae are small membrane invaginations present in endothelial cells, fibroblasts and less abundantly, in cardiomyocytes^1–4^. Caveolin-1 (Cav1) is the main structural protein of the caveolae^5^, as *Cav1* deletion in mice diminishes caveolae formation^6–8^. Similarly, the caveolae associated protein Cavin1, is essential for caveolae formation, because its genetic deletion leads to loss of caveolae^9^. Caveolae participate in multiple cellular processes, including lipid homoeostasis and signal transduction^1,10,11^. In particular, Cav1 interacts directly with Transforming growth factor β receptor-1 (TGFβR1), blocking Smad complex nuclear translocation and, consequently, inhibiting transcriptional activation^12^. Furthermore, caveolae are involved in mechanoprotection, as they deliver the extra membrane needed for cells to buffer mechanical forces through rapid disassembly and flattening^13,14^. Physiologically, caveolae protect mouse cardiac endothelial cells from rupture caused by increased cardiac output^10^. Likewise, caveolae safeguard zebrafish skeletal muscle cells from rupture after vigorous activity^15^ and maintain notochord’s integrity^16,17^.

Genetic inactivation of *Cav1* in the mouse results in cardiac remodelling. Right ventricle dilatation and left ventricle hypertrophy are among the various cardiac defects associated with loss of caveolae^1,8,18^. Additionally, *Cav1* mutant mice show defective heart function, including decreased systolic and diastolic function^1,8,18,19^, which is exacerbated after myocardial infarction^20,21^. Cardiac insult in *Cav1* mutant mice also leads to aberrant fibrosis, mediated by increased Smad2/3 phosphorylation and macrophages infiltration^21,22^. In zebrafish, the *cav1* gene generates two protein-coding transcripts, *cav1a* and *cav1b*, with the Cav1b protein being shorter, as it lacks the first 31 amino acids^23^. Resection of the ventricular apex in hearts of *cav1a* mutant zebrafish leads to regeneration defects 30 days post amputation (dpa), because of decreased cardiomyocyte proliferation and increased fibrosis in the amputation plain^24^. In addition, inactivation of both *cav1a* and *cav1b* transcripts, results in regeneration defects after ventricular resection only in heterozygous animals^24^.

Here, we have generated *cav1-KO* zebrafish and investigated the importance of Cav1 and caveolae in the mechanical properties of the cardiac tissue and in regeneration. We used the cryoinjury model of heart regeneration that leads to extensive fibrotic response, since Cav1 regulates negatively TGFβ pathway^12^. We found that while the absence of Cav1 does not affect cardiac regeneration, *cav1-KO* hearts show a transient decrease in cardiomyocyte proliferation during this process. Using atomic force microscopy (AFM)-force spectroscopy measurements^25^, we detected a substantial reduction in cardiac elasticity in *cav1-KO* animals. Accordingly, epicardial cells and cells of the cortical zone in *cav1-KO* hearts lacking caveolae are stiffer than wild type (WT) counterparts. Furthermore, *cav1-KO* hearts showed a severe ventricular dysfunction, underscoring the role of caveolae in the mechanical properties and homeostasis of the heart.

## Results

### Caveolin-1 expression in the intact and regenerating zebrafish heart

**Figure 1:**
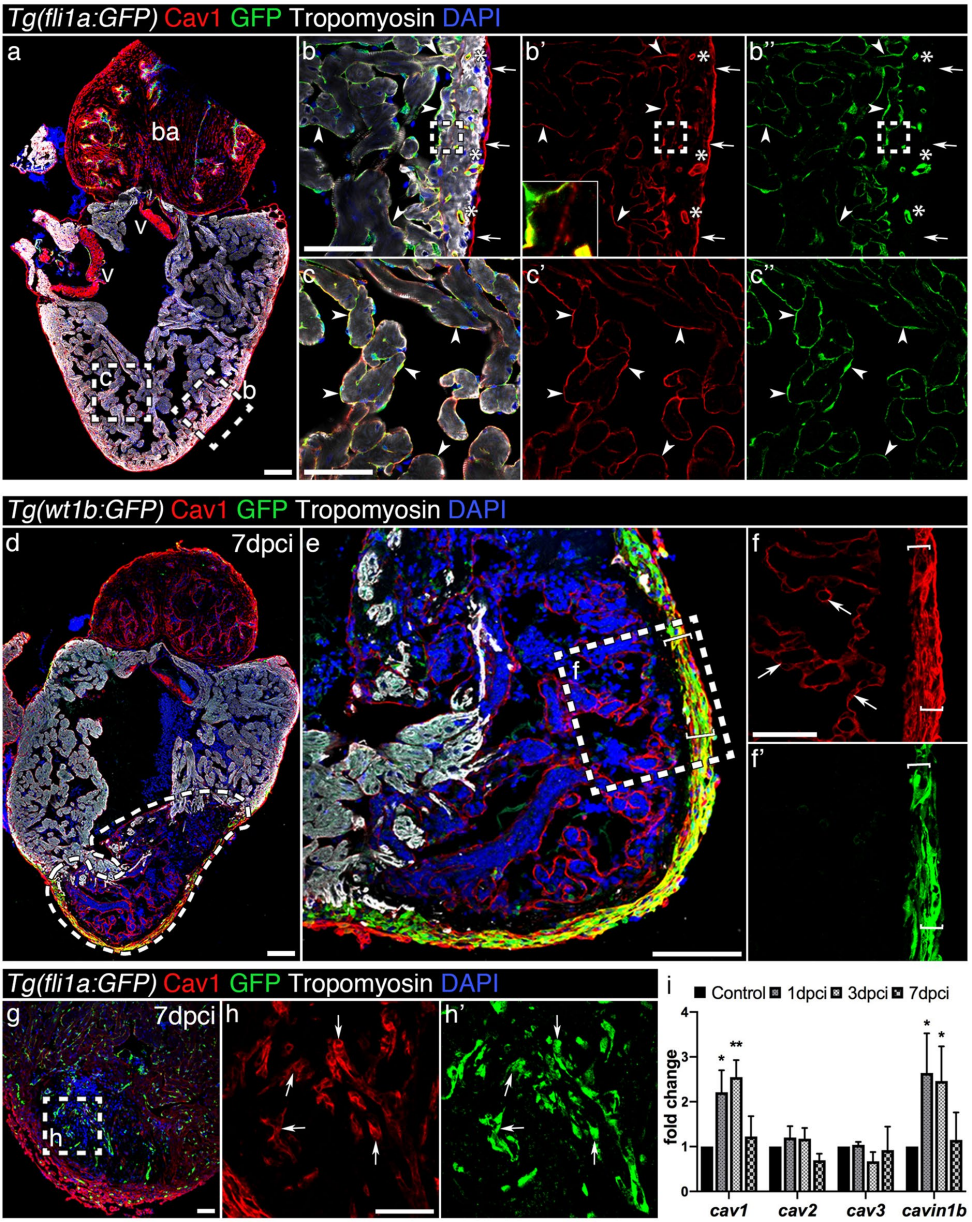
Caveolin-1 is expressed in the endothelium, endocardium and epicardium of the intact and injured adult zebrafish heart. (**a**) Immunofluorescence staining of Cav1 and Tropomyosin (cardiomyocytes) in an intact *Tg(fli1a:GFP)* heart. ba, bulbus arteriosus; v, valves. (**b**–**c′′**) Cav1 immunoreactivity in the epicardium (arrows) overlaps with GFP in the endothelium (asterisks) and endocardium (arrowheads). Cav1 is also expressed in the zone between the cortical and trabecular cardiomyocytes (insert in **b′**). (**d**–**f′**) Cav1 immunostaining in 7 dpci *Tg(wt1b:GFP)* heart. The dashed area in (**d**) marks the injured area; Cav1 is expressed in the activated epicardium (**e**–**f′** brackets) and endocardium (**f**, arrows) upon injury. (**g**–**h′**) Immunolabelling of Cav1 in a 7 dpci *Tg(fli1a:GFP)* heart. (**h**–**h′**) Arrows indicate Cav1^+^ endocardial cells. Scale bars: 100 μm in (**a**), (**d**), (**e**) and 50 μm in other panels. (**i**) qPCR analysis of caveolae-related genes during regeneration. Mean ± s.d., Brown-Forsythe and Welch ANOVA tests, *P < 0.05, **P < 0.01.

We began our analysis by examining Cav1 expression in intact hearts. We used the *Tg(fli1a:GFP)* line^26^, which expresses GFP in the endocardium and endothelium, and stained with antibodies against Cav1 and tropomyosin (Fig. 1a). Robust Cav1 expression was detected in the vasculature (asterisks in Fig. 1b, b′, b′′) and in the endocardium (arrowheads in Fig. 1b, b′, b′′ and c, c′, c′′). Strong expression was also found in the epicardium (arrows in Fig. 1b, b′, b′′), in the bulbus arteriosus and in the valves (Fig. 1a). Additionally, Cav1 expression was detected in the area between the cortical and trabecular myocardium (dashed area in Fig. 1b, b′, b′′ and inset in Fig. 1b′). We then analysed Cav1 expression in the regenerating zebrafish heart after cryoinjury (Fig. 1d). We used the *Tg(wt1b:GFP)* line that expresses GFP in the epicardium upon injury^27^. Seven days post cryoinjury (dpci), Cav1 was strongly expressed in epicardial cells (Fig. 1e, f, f′ brackets) covering the injured site, overlapping with GFP. High expression was also detected in the endocardium within the injured area (Fig. 1f, arrows). To confirm these observations, we utilised the *Tg(fli1a:GFP)* line and found that Cav1 was expressed in GFP^+^ endocardial cells invading the damaged tissue (Fig. 1g, h, h′, arrows). We also surveyed the expression of caveolae-related genes during heart regeneration by quantitative (q)PCR (Fig. 1i). *cav1* and *cavin1b* were upregulated after injury, in contrast to *cav2* and *cav3* whose expression remained stable. These results show that Cav1 is expressed in the endocardium, endothelium and epicardium of the intact heart, three cell types that are activated during regeneration^28–31^. Also, upon injury, Cav1 expression is strongly increased in epicardial cells surrounding the injured site, and in the endocardium invading the injured area.

### Generation of *cav1*-KO zebrafish by CRISPR/Cas9 editing

**Figure 2:**
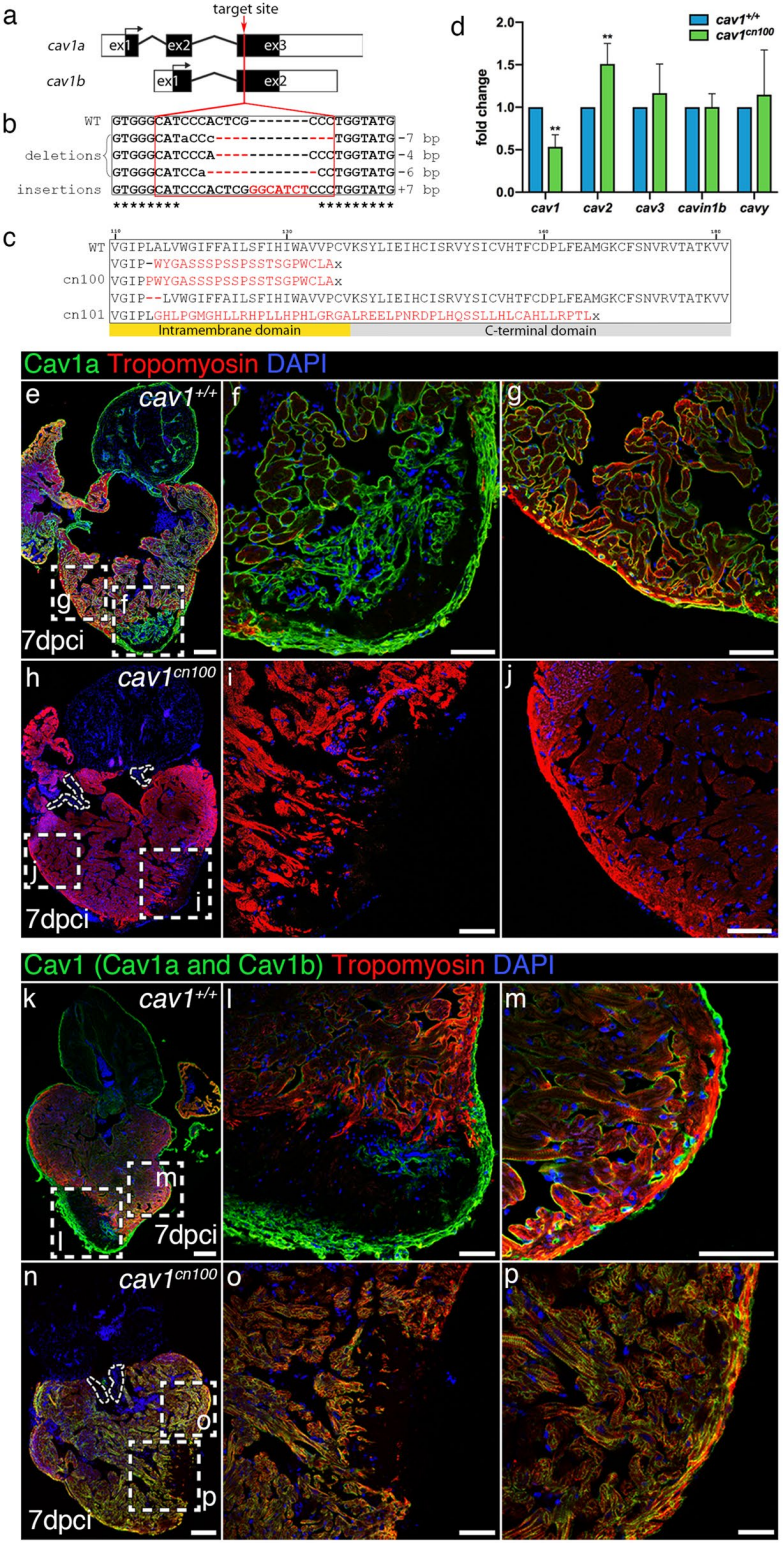
Generation of *cav1*-KO by CRISPR/Cas9 editing and transcriptional analysis. (**a**) Schematic representations of the two *cav1* transcripts—*cav1a* and *cav1b*. Target site of the guide RNA is indicated by a red arrowhead. ex, exon. (**b**) Identified genetic mutations. Red dashed lines indicate deletions, red uppercase letters insertions, and black lowercase letters silent mutations. bp, base pairs. (**c**) Cav1 domain organisation and the predicted effect of the mutations on the protein. The novel amino acids are in red, x indicates a stop codon. (**d**) qPCR analysis of caveolae-related genes in two-day post fertilisation embryos. Mean ± s.d., t-test, **P < 0.01. (**e**–**j**) Immunostaining of Cav1a in 7 dpci hearts. (**f**, **g**, **i**, **j**) Higher magnification of the dashed boxes in (**e**) and (**h**), respectively. (**k**–**p**) Cav1 (Cav1 and Cav1b) immunolabelling of 7 dpci hearts. (**l**, **m**, **o**, **p**) Higher magnification of the dashed boxes in (**k**) and (**n**), respectively. Dotted areas in (**h**) and (**n**) mark the valves in the mutants. Scale bars: 100 μm in (**e**), (**h**), (**k**), (**n**); 50 μm in other panels.

We generated *cav1-KO* zebrafish to study the role of Cav1 and caveolae in heart homeostasis and regeneration*.* We used CRISPR/Cas9 editing to target the third exon of *cav1*, which corresponds to the C-terminal region of the protein (Fig. 2a). The zebrafish *cav1* gene generates two transcripts—*cav1a* and *cav1b*—sharing the majority of the coding sequences, with *cav1a* giving rise to 31 amino acids longer protein^23^. We were able to introduce and identify mutations in the *cav1* locus, with the majority of them being small deletions (Fig. 2b). The predicted effect on the protein was an open reading frame shift that would lead to an amino acid change and the generation of a premature stop codon (Fig. 2c). We selected the *cav1*^*cn100*^ mutation, which showed significantly decreased *cav1* expression, increased *cav2* expression, but with no effect on *cav3*, *cavin1b* or *cavy* transcription (Fig. 2d). Mutant embryos had no morphological abnormalities, developed normally and were fertile (data not shown). We next investigated Cav1 expression by labelling 7 dpci WT and mutant samples with an antibody against Cav1a (Fig. 2e–j and Supplementary Fig. S1a). The strong Cav1a signal in the epicardium, endocardium, endothelium, bulbus arteriosus and valves (Fig. 2e–g) was lost in *cav1*^*cn100*^ hearts (Fig. 2h–j). We repeated this analysis with an antibody that recognises both Cav1 proteins, Cav1a and Cav1b (Fig. 2k–p and Supplementary Fig. S1b). Normal Cav1 expression in epicardium, endocardium and endothelium (Fig. 2k–m) was absent in *cav1*^*cn100*^ mutants (Fig. 2n–p), indicating the loss-of-function (LOF) nature of the mutation. Cavin1 is also an essential component of caveolae^32^ and deletion of *Cav1* diminishes Cavin1 expression in mice^33^. We therefore asked whether Cavin1 was affected by Cav1 loss. Examination of 7dpci *cav1*^+*/*+^ cryoinjured hearts revealed strong Cavin1 expression in the epicardium, in cardiomyocytes adjacent to the injured area, in the bulbus arteriosus and in the valves (Supplementary Fig. S2). Cavin1 expression was decreased in *cav1*^*cn100*^ hearts (Supplementary Fig. S2). Specifically, Cavin1 was absent in the valves, whereas its expression was greatly reduced in the bulbus arteriosus (Supplementary Fig. S2e) and in cardiomyocytes within the proliferative zone (Supplementary Fig. S2g, g′). Taken together, these results show that the *cav1*^*cn100*^ mutation leads to the loss of Cav1 and to the reduced expression of Cavin1 in cryoinjured hearts.

### Loss of caveolae in *cav1*^*cn100*^ mutant hearts

**Figure 3:**
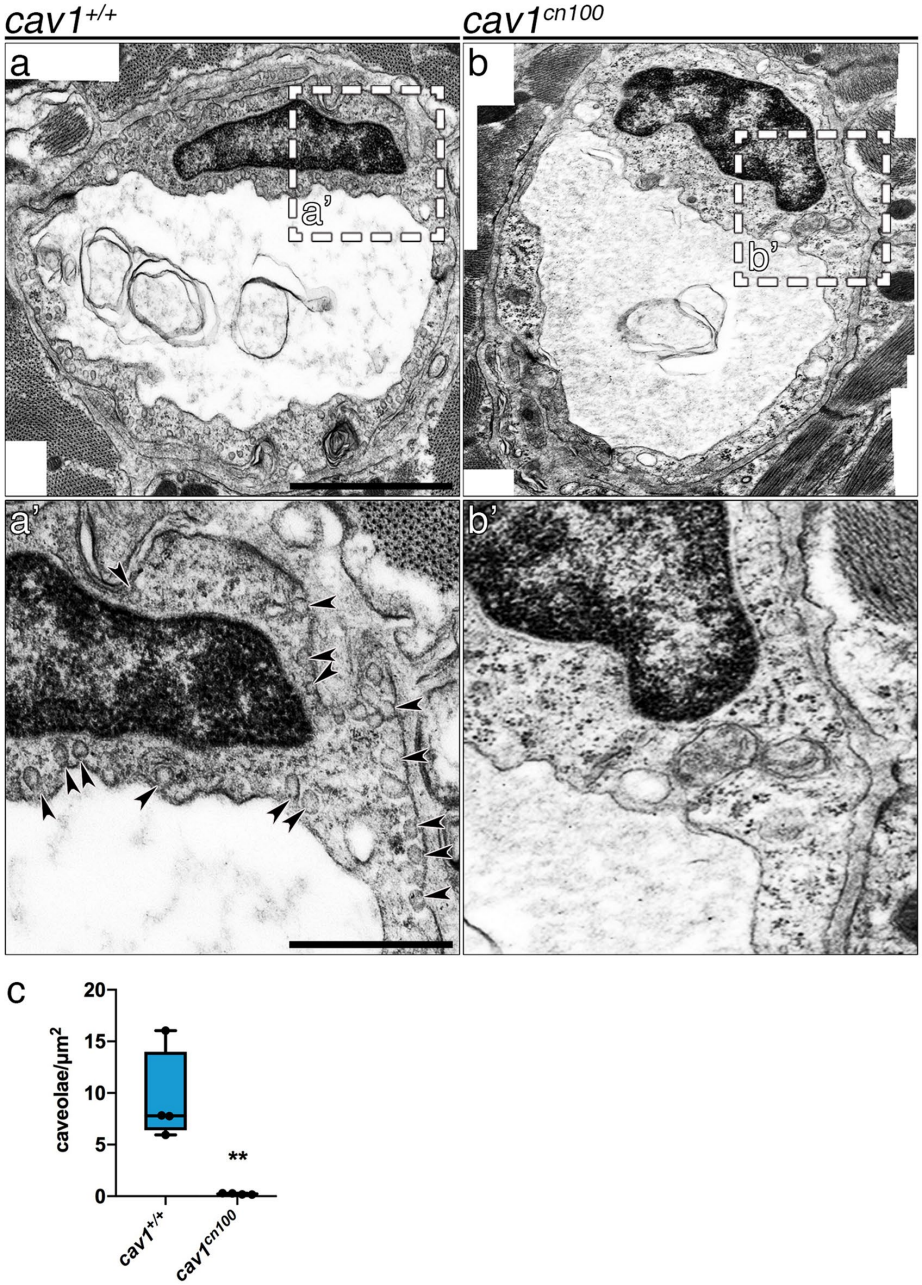
Loss of caveolae in *cav1*^*cn100*^ hearts. TEM images of *cav1*^+*/*+^ (**a**, **a′**) and *cav1*^*cn100*^ (**b**, **b′**) endothelial cells from the coronary vasculature. (**a′**, **b′**) Higher magnification of the dashed areas in (**a**) and (**b**). Arrowheads indicate membrane-bound caveolae. Scale bars: 1 μm in (**a**), (**b**); 0.5 μm in (**a′**), (**b′**). (**c**) Caveolae number per μm^2^ of endothelial cell. n_WT_ = n_cn100_ = 4, t-test, **P < 0.01.

Expression of *Cav1* in a system without endogenous Cav1 expression leads to de novo caveolae formation^5^, whereas deletion of *Cav1* results in loss of caveolae^6–8^. Hence, we asked whether *cav1*^*cn100*^ mutants could form caveolae. To address this, we analysed *cav1*^+*/*+^ and *cav1*^*cn100*^ hearts by transmission electron microscopy (TEM) (Fig. 3). We found caveolae in abundance in *cav1*^+*/*+^ hearts (Fig. 3a, a′), and the membrane of the endothelial cells was packed with caveolae-invaginations (Fig. 3a′, arrowheads). By contrast, *cav1*^*cn100*^ hearts were deprived of caveolae in the coronary vasculature of the cortical layer (Fig. 3b,b′,c). No membrane-bound caveolae were detected, indicating the complete loss of caveolae in *cav1*^*cn100*^ mutant hearts.

### Response of caveolae-depleted hearts to injury

**Figure 4:**
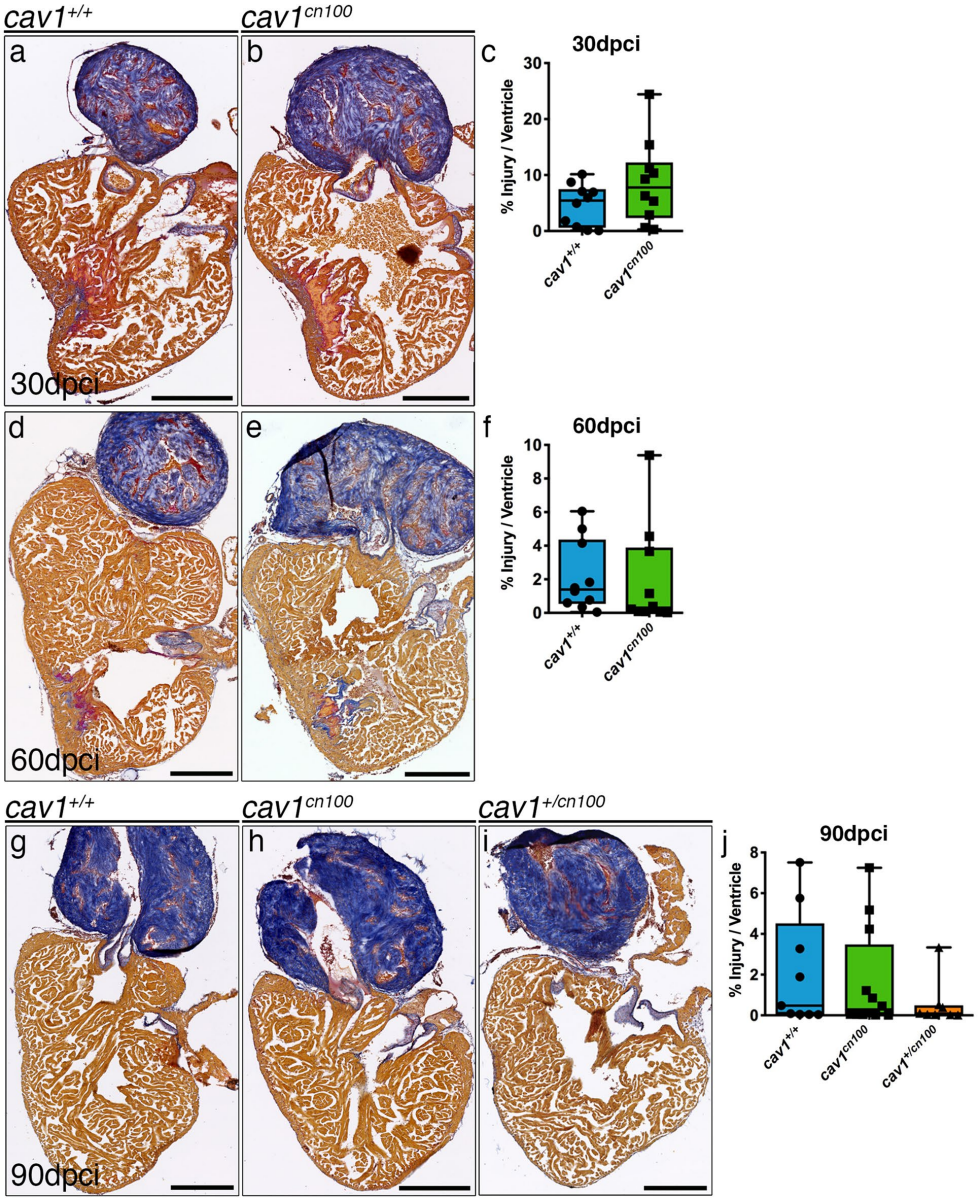
Heart regeneration is unaffected in *cav1*^*cn100*^ mutants. (**a**–**j**) *cav1*^+*/*+^ and *cav1*^*cn100*^ hearts were cryoinjured and harvested 30 (**a**, **b**), 60 (**d**, **e**) or 90 dpci (**g**, **h**), and processed for AFOG staining, which labels collagen in blue, fibrin in red and healthy myocardium in brown. (**i**) heterozygous *cav1*^+*/cn100*^ heart, 90 dpci. (**c**, **f**, **j**) Quantification of the injury site. Injuries were quantified as a percentage of the damaged tissue (collagen and fibrin) to the total area of the ventricle. 30 dpci n_WT_ = n_cn100_ = 10; 60 dpci n_WT_ = n_cn100_ = 10; 90 dpci n_WT_ = 9, n_cn100_ = 12, t-test. Scale bars 250 μm.

We next examined the effects of caveolae loss in adult heart regeneration. We cryoinjured *cav1*^+*/*+^ and *cav1*^*cn100*^ hearts and allowed them to regenerate for 90 days. We then analysed the hearts by Acid Fuchsin Orange-G (AFOG) staining, which labels both the damaged area and the healthy myocardium. We found that homozygous *cav1*^*cn100*^ and heterozygous *cav1*^+*/cn100*^ hearts had regenerated similarly to *cav1*^+*/*+^ hearts 90 dpci (Fig. 4g–j). Cryoinjury results in the formation of a scar tissue that progressively degrades during the course of 90 days^34–36^. Thus, we monitored the regeneration process by analysing injured hearts at 30 and 60 dpci (Fig. 4a–f). *cav1*^*cn100*^ hearts had a similar scar size to that of *cav1*^+*/*+^ controls, both at 30 and 60 dpci.

To further investigate the effect of caveolae loss in heart regeneration, we examined another *cav1* mutant, *cav1*^*cn101*^. Interestingly, expression of *cav1* transcript in the *cav1*^*cn101*^ mutant embryos was unchanged, whereas *cav2* was upregulated (Supplementary Fig. S3a), in accord with our observations in *cav1*^*cn100*^ mutants. In addition, the *cav1*^*cn101*^ mutation had the same effect on protein expression as the *cav1*^*cn100*^ mutation, with a loss of Cav1 expression (Supplementary Fig. S3). Likewise, caveolae were absent in *cav1*^*cn101*^ hearts, similar to our observations for *cav1*^*cn100*^ (Supplementary Fig. S4). We then cryoinjured *cav1*^+*/*+^ and *cav1*^*cn101*^ hearts and examined the regeneration process every 30 days for 90 days (Supplementary Fig. S5). *cav1*^*cn101*^ hearts regenerated normally and we did not detect any differences in the size of the injury at 30, 60 or 90 dpci. These results demonstrate that loss of Cav1 and caveolae do not affect heart regeneration.

### Activation of TGFβ signalling and fibrosis are unaffected in *cav1*^*cn100*^ hearts

Caveolae are involved via Cav1 in the regulation of the TGFβ pathway^12^, a major signalling pathway that controls extracellular matrix (ECM) deposition, and is activated during zebrafish heart regeneration^37^. As both *cav1*^*cn100*^ and *cav1*^*cn101*^ hearts regenerated normally, we focused only on *cav1*^*cn100*^. To address TGFβ activity in *cav1*^*cn100*^ hearts upon cryoinjury, we quantified the nuclear localization of phospho-Smad3 (psmad3), a downstream effector of TGFβ (Supplementary Fig. S6a, b). We used 14 dpci *Tg(fli1a:GFP)* hearts to calculate the proportion of psmad3^+^ nuclei in endocardial cells within the damaged tissue (Supplementary Fig. S6a′, b′, c) and in the cardiomyocytes surrounding the injured site (Supplementary Fig. S6a′′, b′′, d). Analysis revealed that TGFβ signalling was equally active in control and *cav1*^*cn100*^ hearts, in both endocardial cells and cardiomyocytes (Supplementary Fig. S6c, d). Heart cryoinjury in the *Cav1-*KO mouse leads to extensive collagen deposition and cardiac remodelling^22^. Thus, we exploited the AFOG staining protocol to examine the collagen and fibrin content after injury, and we also measured ventricular size (Supplementary Fig. S7a, b). We found no differences between *cav1*^+/+^ and *cav1*^*cn100*^ hearts, neither in collagen deposition nor in fibrin amount in the injury, nor in the size of the ventricle. Furthermore, because hearts of *Cav1-KO* mice show increased interstitial fibrosis^1,6,18,38^, we examined this parameter in intact *cav1*^*cn100*^ hearts using Picrosirius Red to stain collagen fibres. Results showed no difference in interstitial fibrosis between *cav1*^+*/*+^ and *cav1*^*cn100*^ hearts (Supplementary Fig. S7c–e). Thus, loss of Cav1 and caveolae does not affect TGFβ activity or fibrosis in intact or cryoinjured hearts.

### Epicardial, endocardial and cardiomyocyte function in *cav1*^*cn100*^ hearts upon injury

We next examined the behaviour of the different cell types involved in the regeneration process. We first analysed the epicardium and endocardium, where Cav1 is highly expressed 7 dpci (Supplementary Fig. S8). We crossed the *Tg(wt1b:GFP)* line to *cav1*^*cn100*^ to study epicardial proliferation and we found no difference in epicardial proliferation between *cav1*^+*/*+^ and *cav1*^*cn100*^ hearts (Supplementary Fig. S8a, b, c). We then evaluated the abundance of endocardial cells within the damaged tissue by crossing double transgenic fish *Tg(fli1a:GFP)/Tg(myl7:mRFP)* expressing GFP in endocardial/endothelial cells and RFP in the membrane of cardiomyocytes, with *cav1*^*cn100*^ mutants (Supplementary Fig. S8d, e). Three-dimensional volume rendering and analysis of the GFP^+^ cells inside the RFP^-^ area showed that endocardial cells in *cav1*^*cn100*^ hearts populated the injured area similarly to those of *cav1*^+*/*+^ hearts (Supplementary Fig. S8f). These data indicate that epicardial proliferation and the behaviour of endocardial cells are unchanged in *cav1*^*cn100*^ injured hearts.

**Figure 5:**
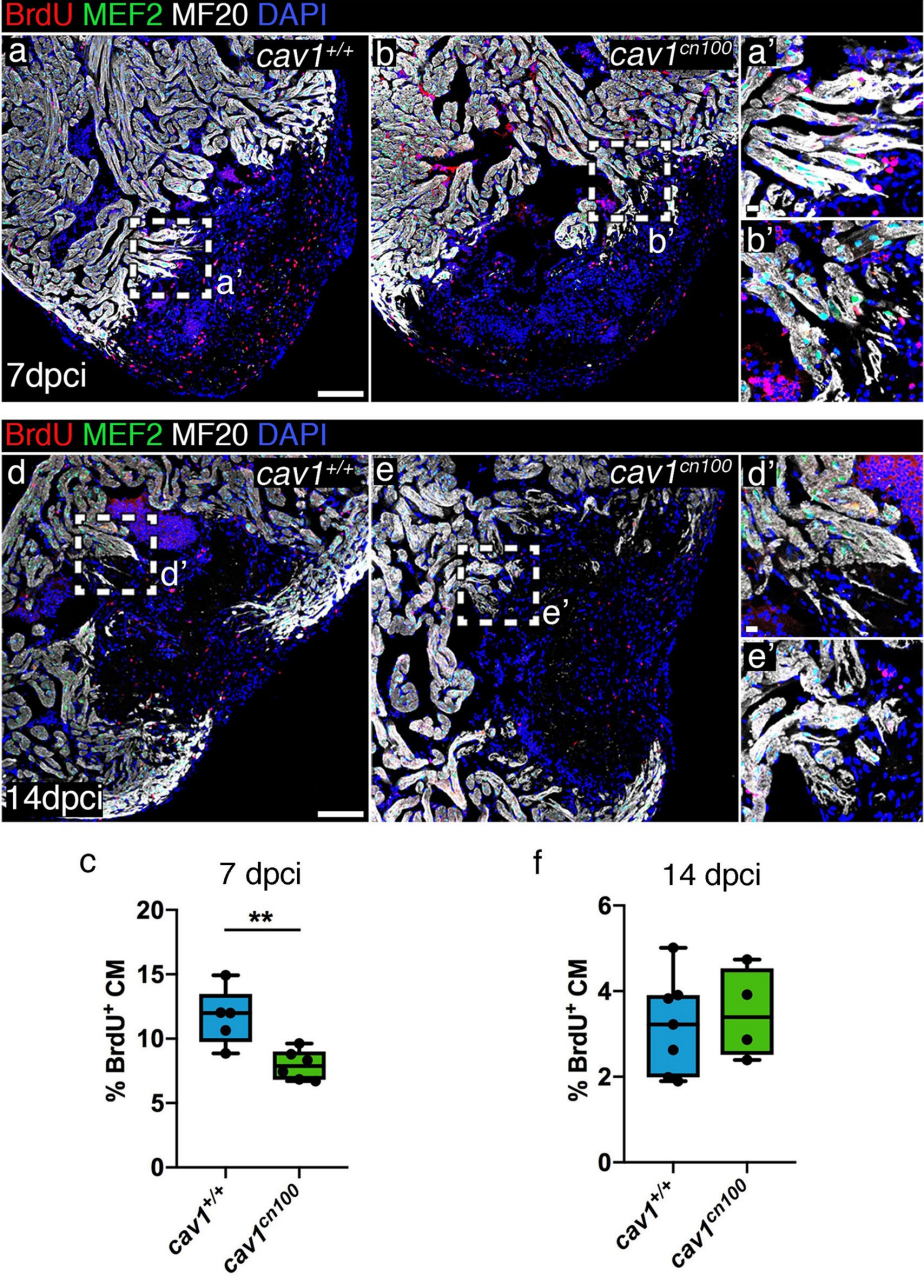
Cardiomyocyte proliferation is transiently reduced upon cryoinjury in *cav1*^*cn100*^ hearts. (**a**, **b**) Immunolabelling of 7 dpci *cav1*^+*/*+^ and *cav1*^*cn100*^ hearts for BrdU, MEF2 and MF20. (**a′**, **b′**) Magnifications of the dashed areas in (**a**) and (**b**). (**c**) Cardiomyocyte proliferation rate was measured by quantifying the BrdU^+^/MEF2^+^ nuclei to the total cardiomyocyte number in a 100 μm radius around the injured area. CM, cardiomyocytes. n_WT_ = 5, n_cn100_ = 6, t-test, **P < 0.01. (**d**, **e**) Immunostaining of 14 dpci *cav1*^+*/*+^ and *cav1*^*cn100*^ hearts for BrdU, MEF2 and MF20. (**d′**, **e′**) Magnifications of the dashed areas in (**d**) and (**e**). (**f**) Quantification of cardiomyocyte proliferation at 14 dpci. n_WT_ = 7, n_cn100_ = 4, t-test. Scale bars: 100 μm in (**a**), (**b**), (**d**), (**e**); 10 μm in (**a′**), (**b′**), (**d′**), (**e′**).

Cardiomyocyte proliferation has been reported to be reduced upon ventricular resection in a *cav1a*-KO zebrafish model^24^. The cardiomyocytes adjacent to the injured area 7 dpci are highly proliferative^39^ and we analysed BrdU incorporation in control and *cav1*^*cn100*^ hearts (Fig. 5a, b, a′, b′). Cardiomyocyte proliferation was significantly lower in *cav1*^*cn100*^ hearts than in *cav1*^+*/*+^ hearts (Fig. 5c). We confirmed this result in the *cav1*^*cn101*^ animals (Supplementary Fig. S9). We then addressed the proliferation status of cardiomyocytes at 14 dpci (Fig. 5d, e). We found that at this time point, *cav1*^*cn100*^ cardiomyocytes proliferated at the same rate as *cav1*^+*/*+^ cardiomyocytes (Fig. 5d′, e′, f). These data indicate that loss of caveolae leads to the transient attenuation in *cav1*^*cn100*^ cardiomyocyte proliferation at 7 dpci, which is normalised by 14 dpci, leading to normal cardiac regeneration at 90 dpci.

### Impaired heart function and cardiac elasticity in *cav1*^*cn100*^ mutants

**Figure 6:**
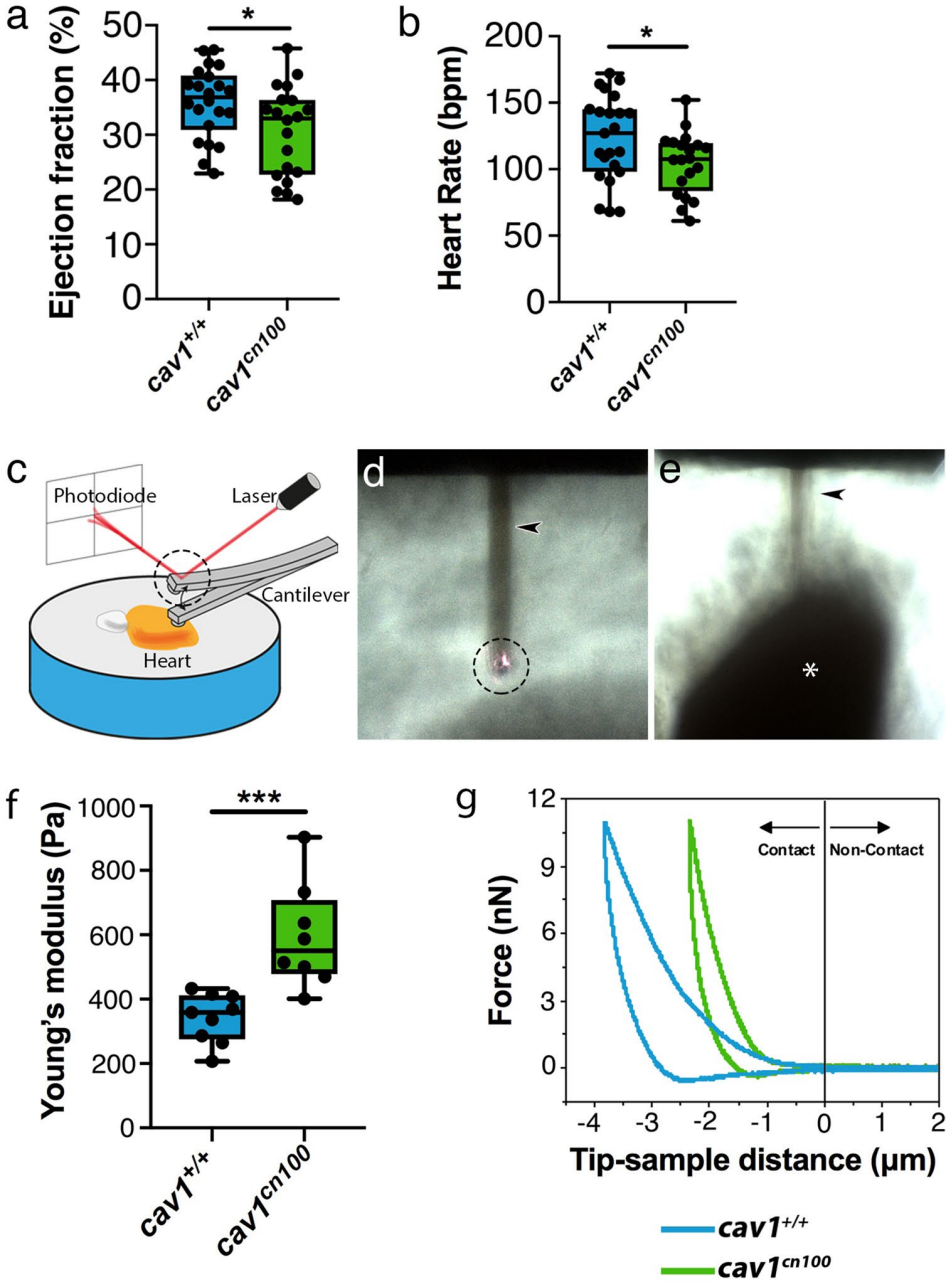
Impaired cardiac function and stiffer heart tissue in caveolae-deprived *cav1*^*cn100*^ mutants. (**a**) Quantification of ejection fraction in *cav1*^+*/*+^ and *cav1*^*cn100*^ hearts. n_WT_ = 22, n_cn100_ = 20. (**b**) Heart rate measurements in *cav1*^+*/*+^ and *cav1*^*cn100*^ animals. n_WT_ = 23, n_cn100_ = 20. t-test, *P < 0.05. (**c**) AFM set-up. Cartoon made with Adobe Illustrator CC2018 (www.adobe.com). (**d**, **e**) Images of the cantilever (arrowhead), the laser beam on top of the cantilever (dashed circle, also in **c**) and the ventricle (asterisk). Images were taken with an inverted optical microscope (AXIO Observer D1; Carl Zeiss, Germany, see Methods). (**f**) Biomechanical characterization of *cav1*^+*/*+^ and *cav1*^*cn100*^ ventricles as measured by AFM-force spectroscopy and expressed in Young’s moduli. n_WT_ = 9, n_cn100_ = 8, t-test, ***P < 0.001. (**g**) Force-distance graph for indentation and retraction of the cantilever over the ventricular surface.

*Cav1*-deficient mice have been reported to show decreased systolic function^1,8^. Therefore, we analysed heart function in our mutants using ultrasound imaging. Echocardiography of eleven months-old adult *cav1*^+*/*+^ and *cav1*^*cn100*^ animals revealed that *cav1*^*cn100*^ hearts were less efficient in pumping blood than control hearts as their ejection fraction was significantly lower (Fig. 6a). Additionally, the heart rate of *cav1*^*cn100*^ animals was lower than in control siblings (Fig. 6b). These findings indicate that caveolae are essential for normal cardiac function.

Caveolae provide protection against mechanical stress^13,14^. Because the biomechanical properties of cells influence the behaviour of tissues^40^ and caveolae are involved in mechanoprotection, we investigated the mechanical response of *cav1*^+*/*+^ and *cav1*^*cn100*^ cardiac tissue to gain insight into the differences observed in cardiac function. We used AFM to determine the response of adult *cav1*^*cn100*^ hearts to force. Freshly isolated hearts were placed on an agarose gel, with the apex of the ventricle oriented towards the direction of the cantilever, and force measurements were taken (Fig. 6c–e). We found that cardiac tissue stiffness was significantly greater in *cav1*^*cn100*^ animals than in control animals (Fig. 6f). Applying the same force, the extent of deformation was 1.5 times greater in control hearts than in *cav1*^*cn100*^ hearts, without causing permanent deformation as the curves returned to zero (Fig. 6g). This result suggests that changes in epicardial and underlying cells, including cortical myocytes, were responsible for the observed difference in stiffness that we detected. That is because the experiments were performed by producing a compressive deformation (indentation) of 1.5 μmin *cav1*^*cn100*^ mutant hearts. The force-distance curves (Fig. 6g) did not show evidence of tissue and/or cell rupture during the deformation. Therefore, the epicardium is a major contributor to the measured stiffness (550 Pa). However, the use of large probes (R = 30 µm) implies that deformation is also transmitted to surrounding regions beyond the 1.5 μm indentation depth. By using bottom-effect corrections^41^ we estimate that the cells within 5–10 μm distance from the contact point will also contribute to the measured stiffness. Membrane invaginations such as caveolae, provide the necessary stretch capacity for cells to buffer the impact of mechanical forces^14^. Hence, the absence of membrane reservoirs, caveolae, due to the loss of the broad and robust expression of Cav1 in the ventricle, results in increased tissue stiffness.

## Discussion

Caveolin-1 is the main structural protein of caveolae, small membrane invaginations involved in signal transduction and mechanoprotection^7,13,14^. Here, we investigated the importance of Cav1 and caveolae in heart homeostasis and regeneration after cryoinjury in zebrafish. We found that while Cav1 expression in the heart is strongly increased upon cryoinjury, its deletion, resulting in loss of caveolae, does not affect heart regeneration. However, loss of Cav1 and caveolae leads to increased stiffness of epicardial cell and cells of the cortical zone, including cardiomyocytes, and impaired cardiac function.

We found that Cav1 is expressed in the endothelium, endocardium and epicardium of intact adult hearts. Also, *cav1* transcription is upregulated following injury and Cav1 is strongly expressed 7 dpci in epicardial cells covering the damaged cardiac tissue, and in the endocardium invading the injured area. We generated *cav1-KO* zebrafish strains to examine the role of Cav1 in the heart. We used two *cav1* mutant alleles, *cav1*^*cn100*^ and *cav1*^*cn101*^. Both mutations are LOF alleles, as they led to the lack of both Cav1a and Cav1b proteins expression. TEM analysis showed that caveolae were completely absent in both *cav1*^*cn100*^ and *cav1*^*cn101*^ hearts, whereas they were abundant in *cav1*^+*/*+^ hearts. Thus, *cav1*^*cn100*^ and *cav1*^*cn101*^ mutations lead to loss of Cav1 and caveolae. This loss also affected Cavin1 protein expression, although transcription was unaffected. The *cav1-KO* hearts lacked Cavin1 in the valves and the overall expression of the protein was decreased, which is in accord with results from the *Cav1-KO* mouse^32,33^.

We investigated the effect of Cav1 and caveolae loss in cardiac regeneration using the cryoinjury model, as it mimics the mammalian myocardial infarction in formation of necrotic myocardium and scar tissue, which in the zebrafish gradually resolves leading to complete tissue regeneration at 90 dpci^34–36^. We found that mutant hearts carrying the *cav1*^*cn100*^ or *cav1*^*cn101*^ LOF mutations in heterozygous or homozygous condition regenerate similar to the WT heart upon cryoinjury and we did not detect differences in injury size at 30 and 60 dpci. These findings were unexpected, as it has been reported that ventricular resection in heterozygous *cav1-KO* zebrafish results in enhanced fibrosis, leading to defects in heart regeneration 30 dpa^24^. The fibrotic response in ventricular resection is minimum, whereas cryoinjury leads to massive fibrosis and to the formation of a transient scar tissue^34–36,42^. However, our data indicate that the formation and resolution of the scar tissue is unaffected by Cav1 loss. Additionally, caveolin-1 negatively regulates TGFβ signalling^12,]^ and loss of murine CAV1 enhances collagen deposition after injury^21,22^. Notably, we found no changes in the quantity of collagen within the damaged tissue between *cav1-KO* and WT hearts, or in TGFβ pathway activation. Likewise, there were no signs of interstitial fibrosis in intact *cav1-KO* hearts, as has been reported in murine *Cav1-KO* models^1,6,18,38^. These results suggest activation of a compensatory mechanism^43^ that buffers Cav1 loss, which in heterozygosity might be induced stronger upon cryoinjury than after resection, in which fibrosis is minimal. Hence, since heart regeneration after ventricular resection lasts up to 60 days^42^, it would be of interest to examine the process at the final time point. Intriguingly, *cav1*^*cn100*^ mutation results in decreased expression of *cav1* transcript, whereas, *cav1* transcription is unchanged in *cav1*^*cn101*^. However, both of our mutations lead to loss of Cav1 protein expression. This could suggest that compensation might be triggered also after protein loss^43^, or that a Cav1-independent TGFβ regulatory pathway may exist in zebrafish heart regeneration.

We detected Cav1 in the epicardium covering the injured area and in endocardial cells invading the damaged tissue. Upon injury, the epicardium is also populated with fibroblasts and macrophages that contribute to fibrosis^44,45^, and loss of *Cav1* in mice is associated with increased fibrosis and macrophage infiltration after cardiac injury^21,22^. The proliferation rate of epicardial cells was unchanged in *cav1-KO* hearts, indicating that these two cell types are unaffected in our model. In addition, endocardial cells have been associated with collagen deposition^31^ and Cav1 was completely lost in these cells. However, endocardial cell abundance was similar between WT and *cav1*^*cn100*^ hearts, suggesting that their proliferation and migration was unaffected. Collectively, these findings further indicate that loss of Cav1 and caveolae does not lead to deregulation of fibrosis in intact or in cryoinjured hearts. Moreover, loss of Cav1 and caveolae triggered a transient but significant decrease in cardiomyocyte proliferation 7 dpci. This was not reflected in defective or even delayed regeneration. Indeed, cardiomyocyte proliferation in mutant fish 14 dpci was similar to that of controls, suggesting that cardiomyocytes were overall able to increase in numbers, populate the damaged area and contribute to normal heart regeneration in *cav1* mutants. In summary, considering that the heart of both of homozygous or heterozygous *cav1* LOF mutants regenerates normally, we can only conclude that Cav1 and caveolae are not required for zebrafish heart regeneration.

Systolic and diastolic functions are also affected in *Cav1*-null mouse hearts^1,8,18,19^ and, in line with this finding, the *cav1*^*cn100*^ line showed impaired cardiac performance, as indicated by the reduced EF and a lower heart rate. As we did not detect cardiac fibrosis, the reduction in cardiac elasticity is a plausible explanation for the cardiac dysfunction in *cav1-KO* fish. Membrane invaginations such as caveolae provide the necessary stretch capacity for cells to buffer the impact of mechanical forces^13,14^. Our AFM-force spectroscopy analysis revealed that *cav1-KO* hearts were significantly stiffer than WT hearts, as cardiac tissue elasticity was decreased almost two-fold after loss of Cav1 and caveolae. The sensitivity of AFM allows us to conclude that the measurements represent the stiffness of both the epicardial cells and the underlying cortical cells, including cardiomyocytes. We have shown that Cav1 is strongly expressed in the epicardium of intact hearts and that its expression is lost in *cav1*^*cn100*^ hearts. Additionally, RNA-seq data show that *cav1* expression is moderate in cortical cardiomyocytes, but significantly higher than in trabecular cardiomyocytes^44^. Caveolae are required for smooth muscle cell contractility^46,47^ and cell contraction results in changes to membrane tension that caveolae buffer^14^. In addition, Cav1 and caveolae regulate RhoA activation (a GTPase protein that regulates the cytoskeleton), which in turn regulates actomyosin contractility^48–50^. Accordingly, these data suggest that loss of Cav1 and caveolae impairs cell elasticity and/or contractility and, consequently, pumping efficiency, which could explain the significant reduction in cardiac performance. Cav1 deficiency in the mouse leads to diverse cardiac phenotypes that are attributed to extensive fibrosis and endothelial loss of Cav1 and caveolae. Our results demonstrate that loss of Cav1 and caveolae result in cardiac stiffening accompanied by reduced cardiac function, suggesting that a global change in the mechanical properties of the heart leads to the cardiac phenotypes observed in Cav1-deficiency models.

## Methods

### Zebrafish husbandry and transgenic lines

Animal studies were approved by the CNIC Animal Experimentation Ethics Committee and by the Community of Madrid (Ref. PROEX 83.8/20). Animal procedures conformed to EU Directive 2010/63EU and Recommendation 2007/526/EC regarding the protection of animals used for experimental and scientific purposes, enforced in Spanish law under Real Decreto 53/2013. Zebrafish were raised under standard conditions at 28 °C as described^51^. Experiments were performed with 5–14-month-old adults.

### CRISPR/Cas9 injections and nature of mutant alleles

We used the oligos *cav1*Fwd CACCGGTGGGCATCCCACTCGCCC and *cav1*Rvs AAACGGGCGAGTGGGATGCCCACC for the generation of *cav1-KO* zebrafish. The oligos were inserted into the pX330-U6-Chimeric_BB-CBh-hSpCas9 vector^52^, which was linearized with BbsI enzyme (New England Biolabs, Ipswich, MA). Primers with the T7 polymerase promoter-specific sequences, *cav1* T7 Fwd TAATACGACTCACTATAGGTGGGCATCCCA and Rvs AAAAAGCACCGACTCGGTGCCA, were used to amplify the guide RNA, which was injected into one-cell state embryos together with Cas9 protein (New England Biolabs). Mutant animals were identified by PCR using the primers: *cav1*Fwd GGCGAGCTTCACCACCTTC and *cav1*Rvs GCTCTTCACGCAAGGCACCA. Both mutant alleles generated and characterized in this study (*cav1*^*cn100*^ and *cav1*^*cn101*^) carry loss-of-function or KO mutations.

### Adult heart cryoinjury

Fish were anesthetised by immersion in 0.04% tricaine (Sigma-Aldrich, St Louis, MO) in fish water and placed on a wet sponge under a stereoscope with the ventral side exposed. The cardiac cavity was opened using microscissors and microforceps, and the pericardium was removed. The ventricle of the heart was exposed and dried and was then touched by a copper-made probe previously immersed in liquid nitrogen^28^. The fish were immediately returned to water to recover.

### Bromodeoxyuridine injection

Adult fish were anesthetised and placed on a wet sponge under a stereoscope. 5-bromo-2′-deoxyuridine (BrdU) was diluted in phosphate-buffered saline (PBS) to 2.5 mg/ml and 30 μl were injected intraperitoneally 24-h before dissection of the hearts.

### Echocardiography

Analysis of cardiac function by echocardiography in eleven months-old adult fish was performed as described^53^. Briefly, the fish were anesthetised by immersion in 60 mM tricaine and 3 mM isoflurane in fish water and transferred to a sponge immersed in the same solution. Images were acquired using the VEVO 2100 system (VisualSonics Inc., Toronto, ON, Canada) with a 50-MHz ultrasound probe. The transductor was immersed in the medium dorsally to the cardiac cavity. The fish were immediately transferred to fresh water to recover after the procedure.

### Histological stains

Acid Fuchsin Orange G-staining (AFOG) and Picrosirius Red staining were performed following standard protocols^42^.

### Immunofluorescence

Sections of paraffin-embedded tissue were permeabilised with PBT (PBS with 0.01% TritonX-100) and washed with PBS before incubation with blocking solution (2% bovine serum albumin, 10% goat serum and 2 mM MgCl_2_ in PBS). Sections were then incubated overnight at 4ºC with the antibodies of choice. Caveolin 1a (Cav1a), this isoform is 31 amino acids longer in the N-terminus region than Cav1b. The Cav1a antibody (Cell Signalling Technology, catalogue #D46G3) was raised against the N-terminus region of Cav1a, including residues surrounding Glu20. Caveolin-1 (BD Transduction Laboratories, catalogue #610059), this antibody recognizes amino acids common to both Cav1a and Cav1b, residues 1–97. PTRF/Cavin1 (Atlas Antibodies AB, Stockholm, Sweden, catalogue #HPA049838), GFP (Aves Labs, Tigard, OR, catalogue #GFP-1010), MEF-2 (Santa Cruz Biotechnology, Santa Cruz, CA, catalogue #sc-313), tropomyosin, MF-20 (MHC, myosin, sarcomere, DSHB, Iowa City, IA, USA), phosho-Smad3 (Abcam, Cambridge, MA, catalogue #ab52903) and BrdU (BD Transduction Laboratories, catalogue #347580). The following day, sections were incubated with the appropriate secondary antibody and mounted after DAPI staining.

### Western blot (WB)

Protein expression analysis by WB was performed in pools of three caudal fins. Samples were incubated in buffer (150 mM NaCl, 25 mM Tris pH 7.5, 1.5 mM MgCl_2_, 1% Triton X—100, 10 mM DTT, phosphatase and protease inhibitors) and sonicated (NESLAB RTE 7). Equal amount of proteins (30 mg) were used for SDS-page electrophoresis. Proteins were transferred to PVDF Immobilon-P (Millipore) and blocked with 5% milk. Primary antibodies against Cav1a, Cav1 and alpha-Tubulin (Thermofisher scientific, catalogue #62204) were diluted in TBS-Tween 0.1%/2% BSA and incubated overnight and then incubated with secondary antibody coupled to horseradish peroxidase (Dako Cytomation). Membranes were incubated with the Immobilon Western HRP substrate (Millipore) and imaged with the Image Quant LAS 4000 mini machine.

### Whole-mount confocal imaging

Analysis of endogenous fluorescence of whole-mount hearts was performed as described^31^. Briefly, hearts were fixed overnight with 2% paraformaldehyde and after several PBS washes the tissues were immersed in 3% agarose. Samples were then incubated in CUBIC I solution^54^ at 37 °C for one week. Agarose blocks containing the hearts were mounted for imaging on a petri dish and approximately 700 μm of the injured ventricle was scanned on a Leica SP8 confocal microscope (Leica Microsystems, Wetzler, Germany) using a 10 × objective.

### Quantitative RT-PCR

Three-to-five biological replicates with three technical replicates of each sample were used for the expression analysis of genes by qPCR using the power SYBR Green Master Mix (Applied Biosystems, Foster City, CA) and the ABI PRISM 7900HT FAST Real-Time PCR System. All measurements were normalised to the expression of 18s^55^. The following primers were used for the qPCR analysis: *18s*Fwd TCGCTAGTTGGCATCGTTTATG, *18s*Rvs CGGAGGTTCGAAGACGATCA, *cav1*Fwd TGGGATGGGGGAATGGAAAC, *cav1*Rvs TAAACGGCGAGTGAGCGTAT, *cav2*Fwd GCGTTTATTGCAGGGATTGT, *cav2*Rvs GGATCACTGGCATCACCAC, *cav3*Fwd CAACGAAGATGTCGTGAAGG, *cav3*Rvs GAGACGGTGAAGGTGGTGTAA, and for *cavin1b* and *cavy* from^17^.

### Electron microscopy

Hearts were fixed in 1% glutaraldehyde/4% paraformaldehyde in PBS overnight. Samples were post-fixed in 1% osmium tetroxide for 60 min and dehydrated through a series of ethanol solutions (30%, 50%, 70%, 95%, 100%) and acetone. After the last dehydration step, samples were incubated in a 1:3, 1:1, 3:1 mixture of DURCUPAN resin and acetone and cured at 60 °C for 48 h. Ultrathin Sections (50–60 nm) were obtained using a diamond knife (Diatome AG, Biel, Switzerland) in an ultramicrotome (Leica Reichert ultracut S. Leica Microsystems) and collected in 200-mesh copper grids. The sections were counterstained with 2% uranyl acetate in water for 20 min followed by a lead citrate solution. Sections were examined with a JEOL JEM1010 electron microscope (Tokyo, Japan) equipped with an Orius SC200 digital camera (Gatan Inc., Pleasanton, CA).

### Image analysis and quantification

To analyse cardiomyocyte proliferation, MEF2-positive nuclei were counted in an area of 100 μm around the injury site using Fiji (ImageJ, NIH). BrdU-MEF2-positive cells were also counted and the % proliferation index was expressed as the MEF2:BrdU-MEF2 ratio. For TGFβ signalling activation, all phospho-smad3-positive cardiomyocyte (100 μm of the injury) or GFP-positive cells (inside the injured area) were counted and normalised to the total number of cardiomyocytes or GFP-positive cells. To quantify the regeneration process, at least three sections, in the middle of the ventricle that contain both of the valves, of each heart were used and the injured area (fibrotic tissue and collagen) was measured using Fiji and expressed as a percentage of the total ventricular area. The 3D analysis of the whole-mount hearts was carried out using Fiji and IMARIS programmes. The volume of the GFP signal inside the injured area (RFP negative) was also measured and presented in relation to the volume of the injury. Electron microscopy images of the plasma membrane of coronary endothelial cells were taken at 50,000 × magnification. Uncoated membrane invaginations of 40–90 nm size were counted^17^ and expressed as density per μm^2^ of the perinuclear area. Two endothelial cells per three sections of the same heart were examined. Fiji was used to calculate the perinuclear area and for caveolae identification.

### Atomic force microscopy (AFM)-force spectroscopy

Adult zebrafish were sacrificed by immersion in 0.16% tricaine and the heart was dissected. The atrium was removed and the ventricle was placed horizontally atop a 4% agarose gel immersed in PBS with 0.1 M KCl to arrest the heartbeat uniformly. AFM-force spectroscopy experiments were performed with a JPK Nanowizard III microscope (JPK Instruments, Berlin, Germany) coupled with an inverted optical microscope (AXIO Observer D1; Carl Zeiss, Germany) and equipped with Plateau-CONT-SPL cantilevers (Nanosensors, Neuchatel, Switzerland), with a nominal spring constant of 0.02–0.77 N/m and a spherical tip shape (R = 30 µm). The actual spring constant of the cantilever was determined using the thermal noise method as implemented in the AFM software. Force-distance curves (FDC) were acquired to determine Young’s modulus of the zebrafish heart apex. The tip-sample distance was modulated by applying a triangular waveform^16^. The tip velocity was set to 10 µm/s and the amplitude to 15 μm. The maximum force exerted on the heart apex during a single FDC was of 11 nN. For each zebrafish heart, a complete sequence of 375 FDC was performed. These FDC were distributed in three different areas of 100 × 100 µm^2^ several hundreds of microns apart. In each area, 125 FDC were measured (all along the zebrafish apex). To determine the contact point, we used a ratio of variances protocol. Young’s modulus was obtained by fitting a section of the force-distance curve (approach semi-cycle of the whole FDC) with a Hertz model for spherical indenters.

### Statistical analysis

Sample sizes, statistical tests and P-values are specified in the figure legends and were determined with GraphPad Prism software (GraphPad Software Inc., San Diego, CA). Statistical tests were two-tailed. *P* values below 0.05 were considered of statistical significance.

## Supplementary information

Supplementary Information.
