# Supplementary material for: Loss of Caveolin-1 and caveolae leads to increased cardiac cell stiffness and functional decline of the adult zebrafish heart
Source: Sci Rep. 2020 Jul 30;10:12816. doi: 10.1038/s41598-020-68802-9 (PMC7393500; doi:10.1038/s41598-020-68802-9)
Supplement: Supplementary file 1 — Supplementary Information. [file 41598_2020_68802_MOESM1_ESM.pdf]

Supplementary Information

Loss of Caveolin-1 and caveolae leads to increased cardiac cell stiffness and functional decline of the adult zebrafish heart

Dimitrios Grivas[1,2], Álvaro González-Rajal[1,3], Carlos Guerrero Rodríguez[4], Ricardo Garcia[4] and José Luis de la Pompa[1,2,*]

[1]Intercellular Signalling in Cardiovascular Development and Disease Laboratory, Centro Nacional de Investigaciones Cardiovasculares Carlos III (CNIC), Melchor Fernández Almagro 3, Madrid 28029, Spain.

[2]Ciber de Enfermedades Cardiovasculares, 28029 Madrid, Spain.

[3]Cell Division Lab, ANZAC Research Institute, Gate 3, Hospital Road, Concord 2139, NSW, Australia.

[4]Materials Science Factory, Instituto de Ciencia de Materiales de Madrid (ICMM), CSIC, 28049 Madrid, Spain.

Supplementary Figure Legends

Supplementary Figure S1. Western blot analysis of Cav1a and Cav1 (Cav1a and Cav1b) protein expression in *cav1*^{cn100} and *cav1*^{cn101} mutants.

(a) Samples from *cav1*$^{+/+}$, *cav1*^{cn100} and *cav1*^{cn101} caudal fins were analysed by Western blot using an antibody against Cav1a (Cell Signalling Technology, catalogue #D46G3). Only the higher molecular weight band was detected in *cav1*$^{+/+}$, indicating the specificity of the antibody against Cav1a. In contrast, Cav1a was lost in *cav1*^{cn100} and *cav1*^{cn101}. Tub, alpha-Tubulin; kDa, kilodalton. (b) WB using an antibody against Cav1 (Cav1a and Cav1b, BD Transduction Laboratories, #610059). Two bands were detected in *cav1*$^{+/+}$, indicating that the antibody recognises both Cav1a and Cav1b, in contrast to *cav1*^{cn100} and *cav1*^{cn101} that both Cav1a and Cav1b were lost.

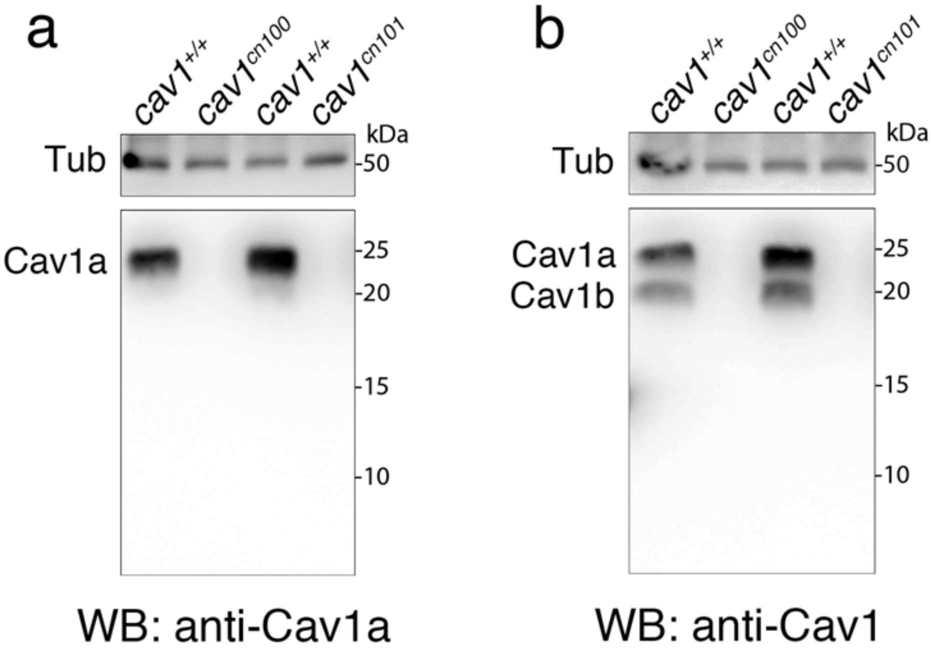
a
b
cav1+/+
cav1cn100
cav1+/+
cav1cn101
kDa
Tub
50
Cav1a
Cav1b
25
20
15
10
WB: anti-Cav1a
WB: anti-Cav1

Supplementary Figure S2. Cavin1b expression in *cav1*^{cn100} hearts

Immunostaining of Cavin1 in 7 dpci *cav1*$^{+/+}$ (a-d´) and *cav1*^{cn100} (e-h´) *Tg(wt1b:GFP)* hearts.
(b, b´) Magnification of selected area in a. (c-d´) Magnifications of the dashed areas in b.
(f, f´) Magnification of selected area in e. (g-h´) Magnifications of the dashed areas in f. Dashed lines in a and e mark the valves. Scale bars: 100 µm in a, b, e, f; 50 µm in other panels.

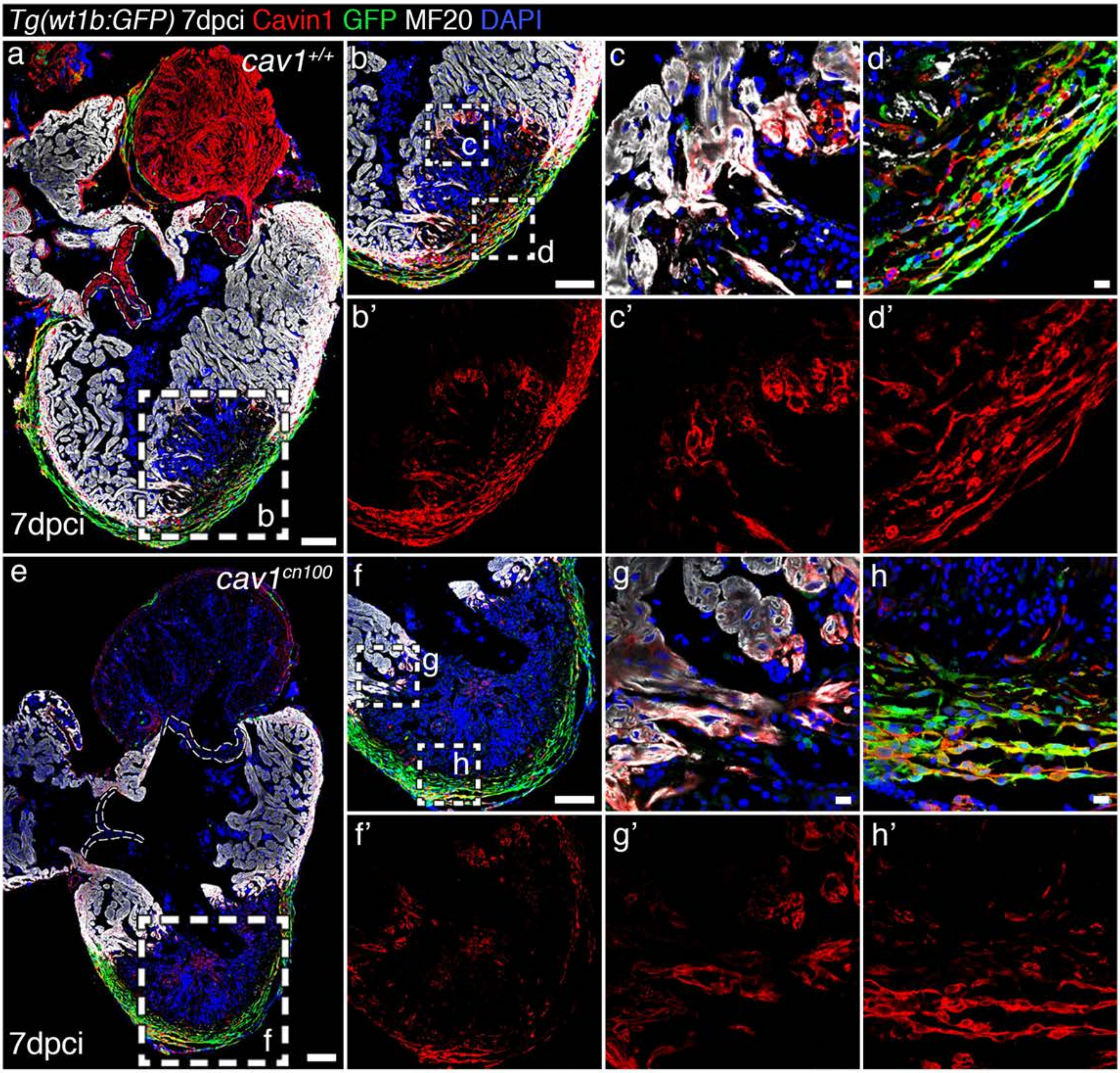

Tg(wt1b:GFP) 7dpci Cavin1 GFP MF20 DAPI
a
cav1+/+
b
c
d
7dpci
b'
c'
d'
e
cav1cn100
f
g
h
7dpci
f'
g'
h'

Supplementary Figure S3. qPCR and Cav1 expression analysis in *cav1^{cn101}* mutants

(a) Relative expression of caveolae-related genes by qPCR in *cav1^{cn101}* embryos. mean±s.d. t-test, ***P<0.001.

(b-m) Immunostaining of 7 dpci *cav1$^{+/+}$* and *cav1^{cn101}* hearts with an antibody against Cav1a (b-g) or Cav1 (Cav1a and Cav1b; h-m). Scale bars: 100 µm in b, e, h, k; 50 µm in other panels.

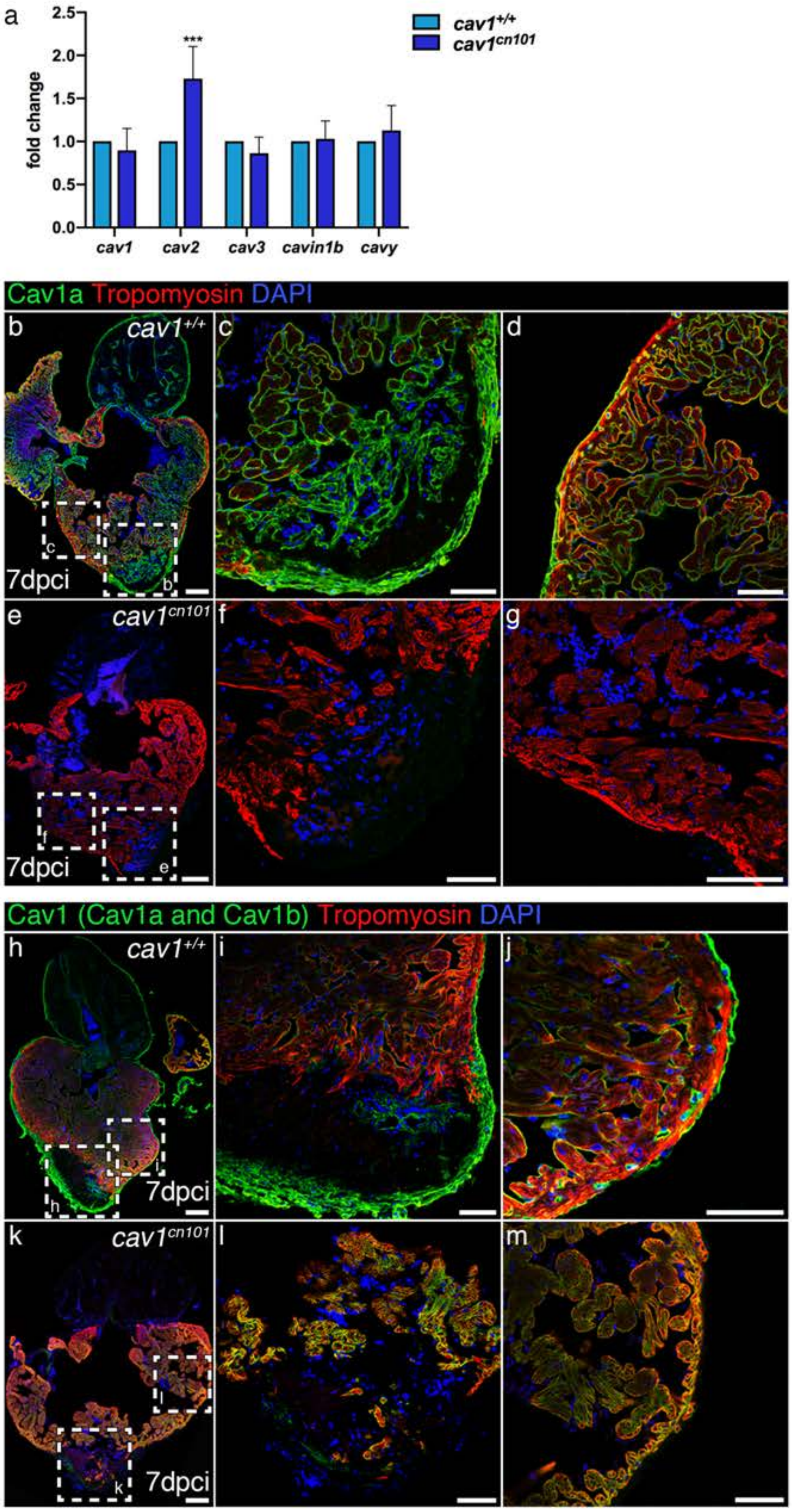
a
fold change
2.5
2.0
1.5
1.0
0.5
0.0
***
cav1
cav2
cav3
cavin1b
cavy
cav1+/+
cav1cn101
Cav1a Tropomyosin DAPI
b
cav1+/+
c
d
7dpci
e
cav1cn101
f
g
Cav1 (Cav1a and Cav1b) Tropomyosin DAPI
h
i
j
k
l
m

Supplementary Figure S4. Caveolae loss in *cav1*^{cn101} hearts

(a-b′) TEM images of coronary vasculature of the cortical layer in *cav1*$^{+/+}$ and *cav1*^{cn101} hearts.

(a′, b′) higher magnifications of the dashed areas in a and b; arrowheads indicate membrane-bound caveolae. Scale bars: 1 µm in a, b, and 0.5 nm in a′ and b′.

(c) Quantification of caveolae number per µm^2 of coronary endothelium. $n_{WT} = n_{cn101} = 4$, mean±s.d., t-test, ****P<0.0001.

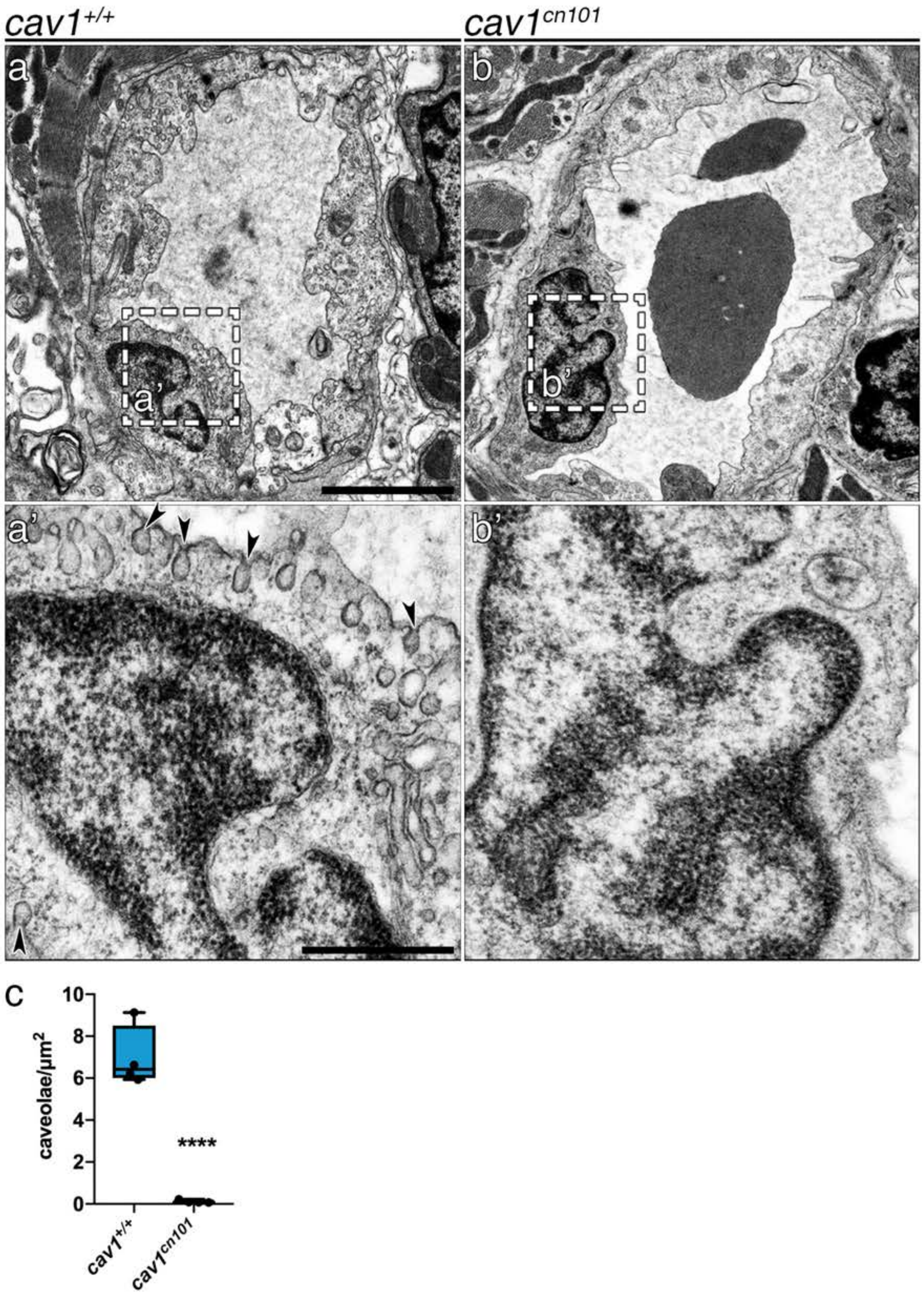
cav1+/+
cav1cn101
a
b
a'
b'
c
10
8
6
4
2
0
caveolae/µm2
****
cav1+/+
cav1cn101

Supplementary Figure S5. Heart regeneration is unaffected in *cav1*^{cn101} mutants

(a-j) AFOG staining of *cav1*$^{+/+}$ and *cav1*^{cn101} hearts after 30 (a, b), 60 (d, e) and 90 (g-i) dpci. Collagen in blue, fibrin in red and healthy myocardium in brown. (c, f, j) The damaged area was quantified as the percentage of the collagen/fibrin area to the total ventricular area. 30 dpci n_{WT}=9, n_{cn100}=7; 60 dpci n_{WT}=8, n_{cn100}=7; 90 dpci n_{WT}=7, n_{cn100}=11. mean±s.d., t-test. Scale bars 250 μm.

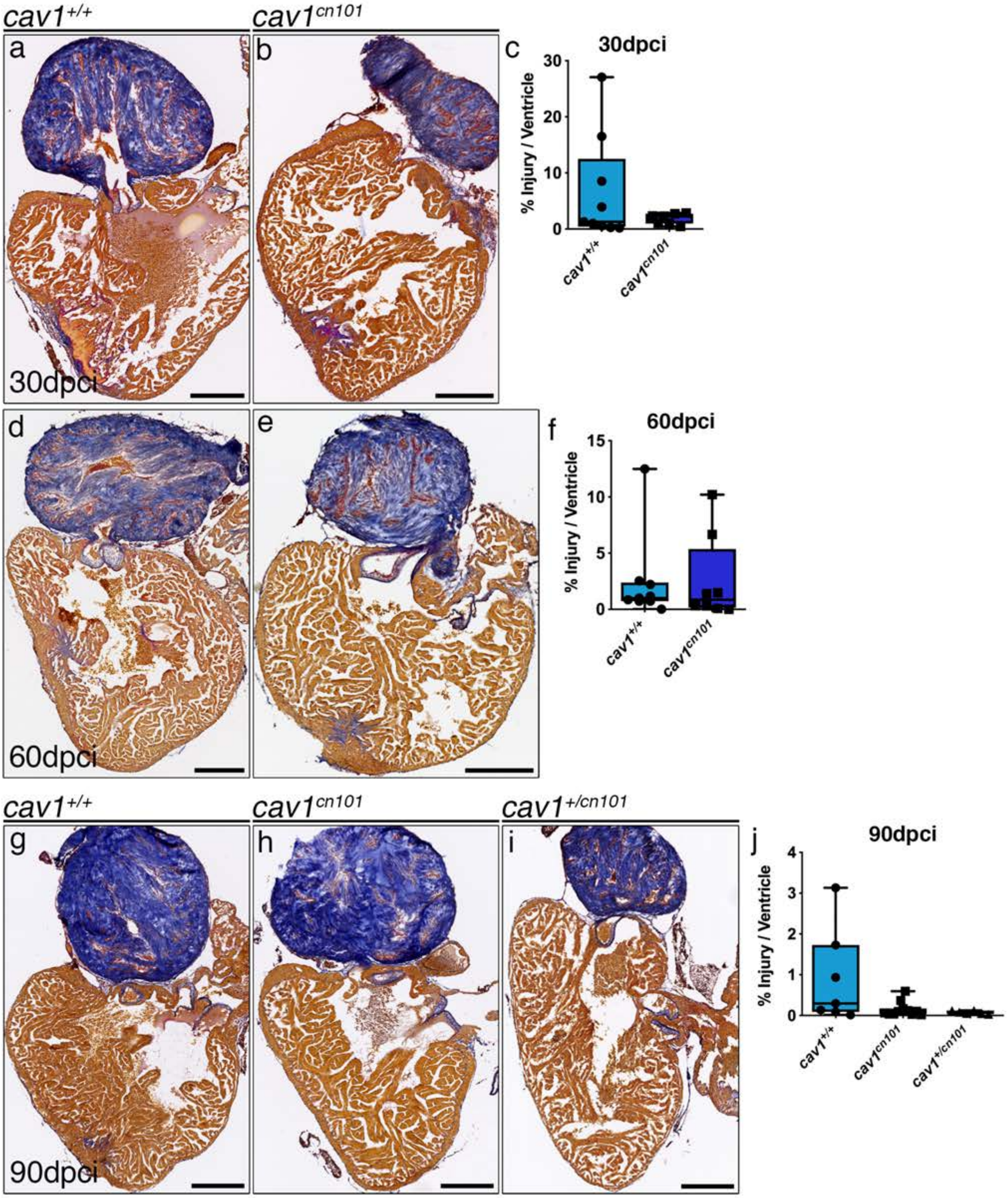

cav1+/+
cav1cn101
cav1+/cn101
a
b
c
d
e
f
g
h
i
j
30dpci
60dpci
90dpci
% Injury / Ventricle
0
10
20
30
5
15
1
2
3
4

Supplementary Figure S6. TGFβ signalling is unaffected in regenerating *cav1^{cn100}* hearts

(a-d) 14dpci *cav1$^{+/+}$* and *cav1^{cn100} Tg(fli1a:GFP)* hearts labelled for psmad3, GFP and MF20. (a′, b′) Magnification of GFP$^+$ endocardial cells marked in a, b. (a′′, b′′) Higher magnification of cardiomyocytes marked in a, b. (c) Quantification of psmad3$^+$/GFP$^+$ in the injured area. t-test. n_{WT} = 7, n_{cn100} = 4. (d) Percentage of cardiomyocytes with psmad3$^+$ nuclei in a 100 µm area surrounding the damaged tissue. CM, cardiomyocytes. t-test, n_{WT} = 7, n_{cn100} = 4. Scale bars 100 µm in a and b; 25 µm in other panels.

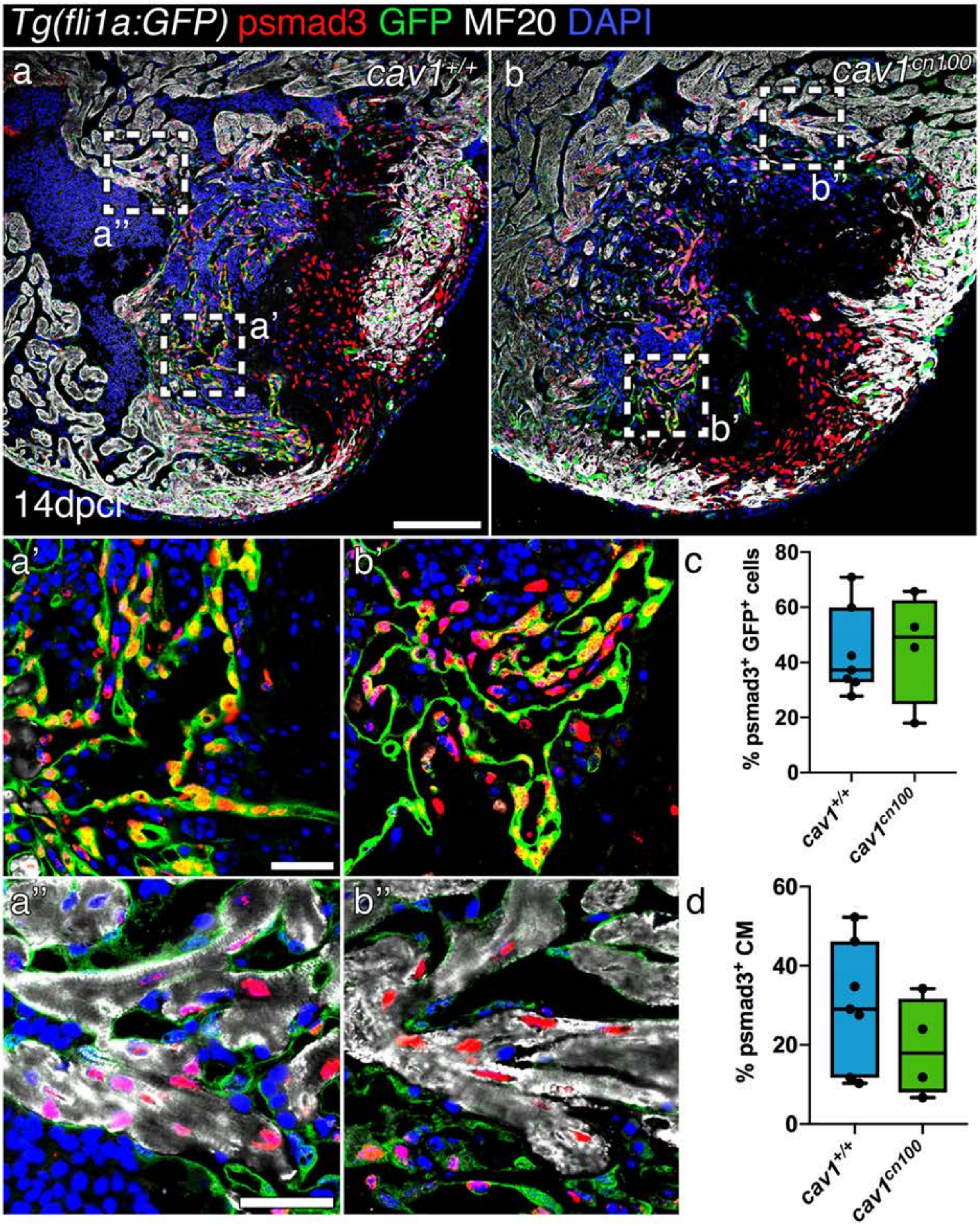
Tg(fli1a:GFP) psmad3 GFP MF20 DAPI
a
cav1+/+
a''
a'
14dpci
b
cav1cn100
b''
b'
a'
b'
a''
b''
c
% psmad3+ GFP+ cells
0
20
40
60
80
cav1+/+
cav1cn100
d
% psmad3+ CM
0
20
40
60
cav1+/+
cav1cn100

Supplementary Figure S7. Analysis of collagen and fibrin in the injured area, ventricular size and interstitial fibrosis of *cav1^cn100^* hearts

(a) Percentages of collagen and fibrin within the injury zone 30, 60 and 90 dpci in $cav1^{+/+}$ and $cav1^{cn100}$ hearts. Two-way ANOVA. n_{WT} 30, 60, 90 dpci = 10, 9, 9; n_{cn100} 30, 60, 90 dpci = 10, 10, 12.

(b) Ventricular size of all hearts analysed by AFOG staining. t-test. $n_{WT} = 29$; $n_{cn100} = 32$.

(c, d) Picrosirius Red staining in intact $cav1^{+/+}$ and $cav1^{cn100}$ hearts. Scale bar 250 µm.

(e) Quantification of the red-labelled fibres in the ventricle. t-test, $n_{WT} = 5$, $n_{cn100} = 6$.

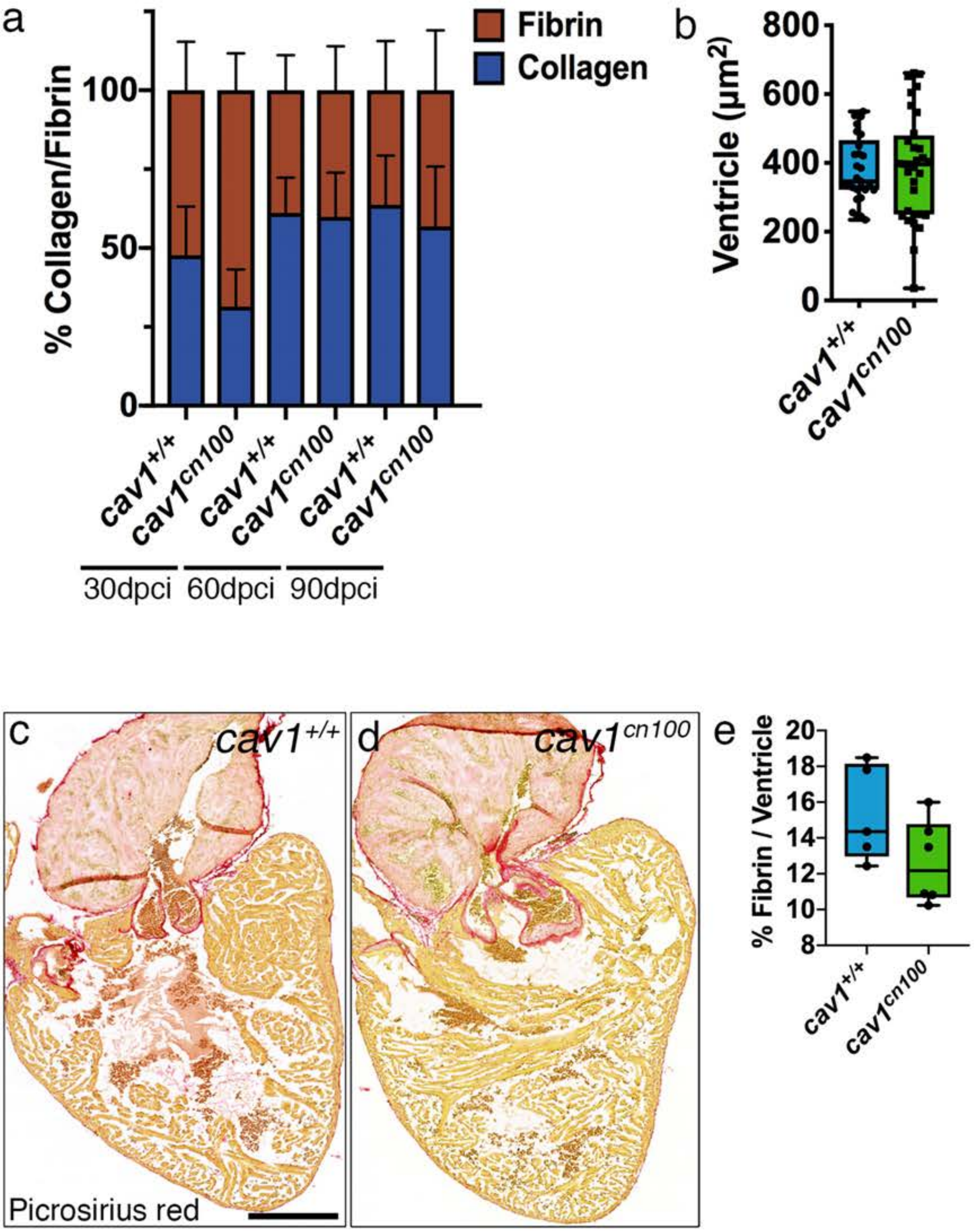
a
Fibrin
Collagen
% Collagen/Fibrin
100
50
0
cav1+/+
cav1cn100
cav1+/+
cav1cn100
cav1+/+
cav1cn100
30dpci
60dpci
90dpci
b
Ventricle (µm2)
800
600
400
200
0
cav1+/+
cav1cn100
c
cav1+/+
Picrosirius red
d
cav1cn100
e
% Fibrin / Ventricle
20
18
16
14
12
10
8
cav1+/+
cav1cn100

Supplementary Figure S8. Epicardial proliferation and endocardial cell function in *cav1^{cn100}* hearts after injury

(a, b) Immunostaining of 7 dpci *Tg(wt1b:GFP)* heart sections labelled for BrdU and GFP.

(c) Percentage of proliferating epicardial GFP^+ cells. t-test, $n_{WT} = 5$, $n_{cn100} = 6$. Scale bar 100 µm.

(d, e) 3D volume rendering of the apical injured site of 7dpci *Tg(fli1a:GFP)/Tg(myl7:mRFP)* hearts. Yellow lines indicate the injured area and heart cartoon the x/y/z axes.

(f) Quantification of the volume of GFP^+ cells inside the RFP^- area. t-test, $n_{WT} = 3$, $n_{cn100} = 4$. Scale bar 300 µm.

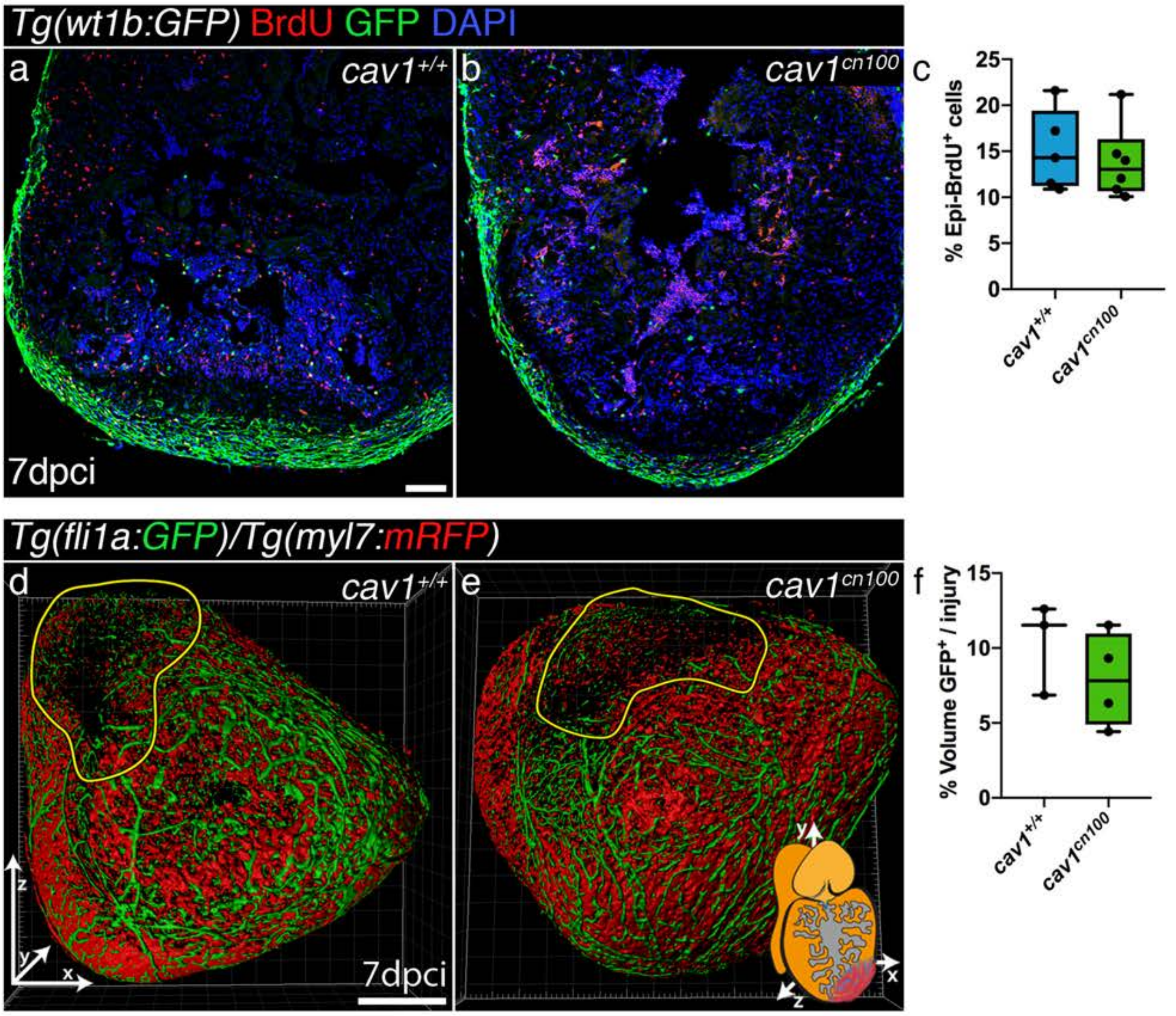
Tg(wt1b:GFP) BrdU GFP DAPI
a
cav1+/+
b
cav1cn100
c
% Epi-BrdU+ cells
25
20
15
10
5
0
cav1+/+
cav1cn100
7dpci
Tg(fli1a:GFP)/Tg(myl7:mRFP)
d
cav1+/+
e
cav1cn100
f
% Volume GFP+ / injury
15
10
5
0
cav1+/+
cav1cn100
z
y
x
7dpci
y
x
z

Supplementary Figure S9. ***cav1**^{cn101}* **cardiomyocyte proliferation upon injury**

(a, b) Immunolabelling of 7 dpci *cav1*$^{+/+}$ and *cav1*^{cn101} hearts for BrdU, MEF2 and MF20. (a′, b′) Higher magnifications of the dashed areas in a and b. Scale bars: 100 µm in a, b; 10 µm in a′, b′.

(c) Percentage of the BrdU^{+} cardiomyocytes to the total number of the cardiomyocytes in a 100 µm area surrounding the damaged tissue. CM, cardiomyocytes. $n_{WT} = 4$, $n_{cn100} = 3$. t-test, *$P<0.05$.

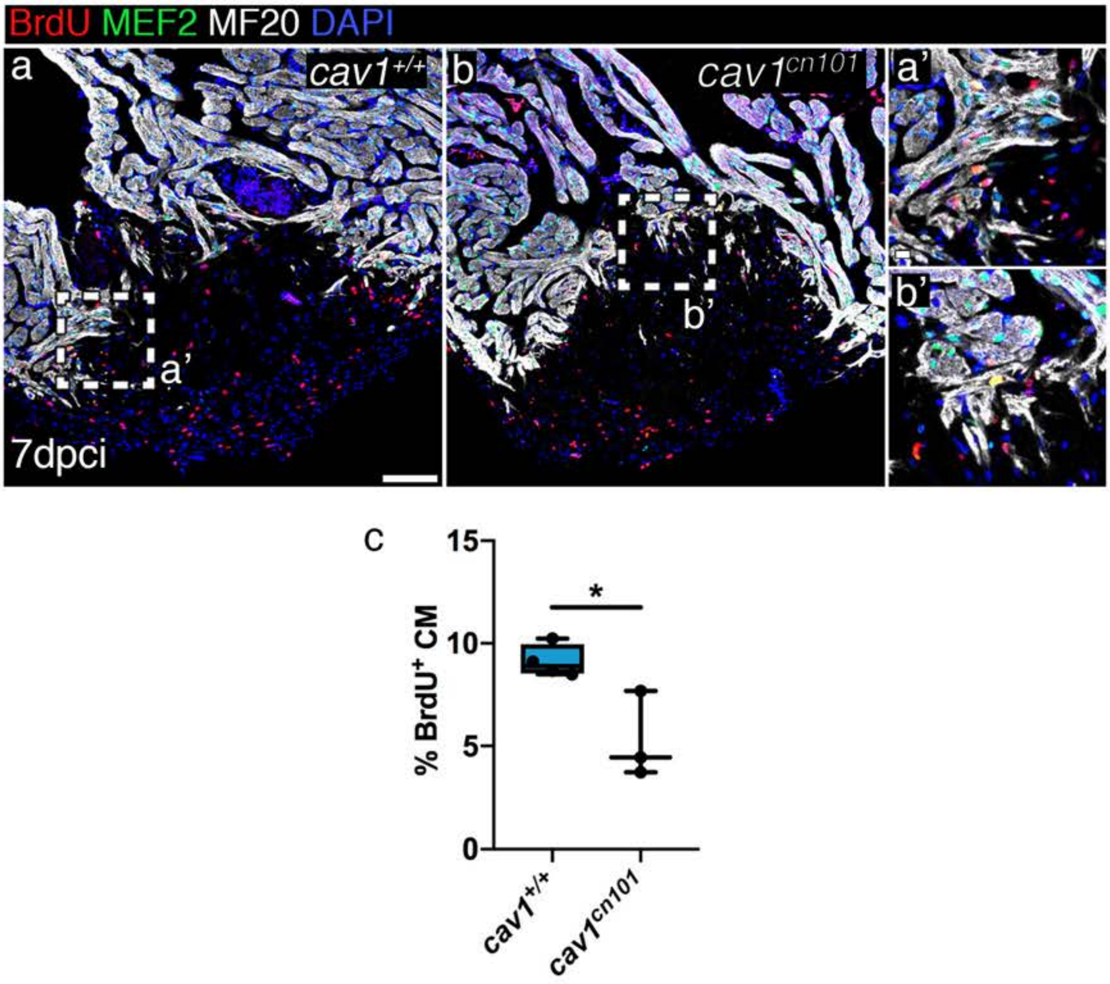

Figure S9_Grivas et al.
